# Photothermally triggered actuation of hybrid materials as a new platform for *in vitro* cell manipulation

**DOI:** 10.1038/ncomms14700

**Published:** 2017-03-13

**Authors:** Amy Sutton, Tanya Shirman, Jaakko V. I. Timonen, Grant T England, Philseok Kim, Mathias Kolle, Thomas Ferrante, Lauren D Zarzar, Elizabeth Strong, Joanna Aizenberg

**Affiliations:** 1 Department of Chemistry and Chemical Biology, Harvard University, Cambridge, Massachusetts 02138, USA; 2 John A. Paulson School of Engineering and Applied Sciences, Harvard University, Cambridge, Massachusetts 02138, USA; 3 Wyss Institute for Biologically Inspired Engineering, Harvard University, Cambridge, Massachusetts 02138, USA; 4 Department of Applied Physics, Aalto University, Espoo 02150, Finland; 5 Department of Mechanical Engineering Massachusetts Institute of Technology, Cambridge, Massachusetts 02139, USA; 6 Department of Materials Science and Engineering and Department of Chemistry, The Pennsylvania State University, University Park, Pennsylvania 16802, USA; 7 Harvard College, Harvard University, Cambridge, Massachusetts 02138, USA

## Abstract

Mechanical forces in the cell’s natural environment have a crucial impact on growth, differentiation and behaviour. Few areas of biology can be understood without taking into account how both individual cells and cell networks sense and transduce physical stresses. However, the field is currently held back by the limitations of the available methods to apply physiologically relevant stress profiles on cells, particularly with sub-cellular resolution, in controlled *in vitro* experiments. Here we report a new type of active cell culture material that allows highly localized, directional and reversible deformation of the cell growth substrate, with control at scales ranging from the entire surface to the subcellular, and response times on the order of seconds. These capabilities are not matched by any other method, and this versatile material has the potential to bridge the performance gap between the existing single cell micro-manipulation and 2D cell sheet mechanical stimulation techniques.

## Abstract (web summary)

Mechanical forces within the cell’s environment play a crucial role in their growth, differentiation and behaviour. Here, the authors develop a photothermal responsive cell culture substrate for the assessment of how cell growth can be affected by manipulating the strain profile of the substrate.

Mechanical forces present in the cell’s natural growth environment have a significant impact on cell gene expression and behaviour^1^,^2^,^3^, and play an important role in regulating the function of tissues and organs, as well as in guiding their development^4^,^5^,^6^,^7^. To understand how forces are translated into different biological outcomes is an active area of research, which has many far-ranging implications in developmental biology, regenerative medicine and cancer research^4^,^5^,^6^,^7^,^8^,^9^,^10^,^11^,^12^. However, the progression of this field is significantly slowed by the limitations of the available methods by which forces can be applied on cells directly in controlled *in vitro* experiments^6^,^11^,^12^,^13^,^14^,^15^. To a great extent, the methods currently in use either sacrifice experimental control in order to limit the invasiveness of the method and better approximate *in vivo* conditions, or else the level of control is increased at the expense of invasiveness. To date, as all of these methods have disadvantages, the method of choice for an experiment is very context dependent. The single cell micro-manipulation techniques, which include micropipette deformation^16^, atomic force microscopy^17^,^18^,^19^ and optical tweezers^20^,^21^,^22^, allow for high-spatiotemporal precision in the application and measurement of forces, but are hampered by some shortcomings including the limited similarity of the applied strain to the types of strains that cells experience *in vivo*, and the inability to scale up to more than one cell at a time^11^,^12^. The techniques for manipulating populations of cells in two-dimensional (2D) cell sheet culture formats, such as magnetic twisting cytometry and flexible membrane devices^23^,^24^,^25^,^26^,^27^,^28^, allow cells to be grown in fairly typical culture conditions and are therefore less invasive than the single cell techniques, but meanwhile suffer from the opposite drawback with respect to scale and selectivity because there is no user control over which cells on the surface are stimulated^11^,^12^. Notably, the techniques that rely on the deformation of flexible membranes that serve as the cell growth surface (Flexcell International, CellScale) have the important advantage that they allow strain to be generated via deformation of the growth surface^11^,^12^,^26^,^27^,^28^, which is a better approximation of strain from tissue distortion^13^,^29^.

The field of active materials offers the possibility of bridging the gap between the more precise single cell techniques and those that manipulate the 2D growth surface, by providing diverse means of controllably deforming defined areas of a surface. Several types of active cell culture materials with impressive capabilities have been reported in the literature. They have exploited the properties of shape-memory polymers (SMPs)^30^,^31^, magnetic particles dispersed in soft deformable matrix materials^32^,^33^, and temperature-sensitive and reversible volume-changing hydrogels such as poly(N-isopropylacrylamide) (PNIPAAm)^34^,^35^. However, none have reached the goal of generating rapid and directional surface reconfiguration and cell deformation in sub-cellular sized regions of the surface that are not pre-determined during the fabrication process^30^,^31^,^32^,^33^,^34^. In addition, while the magnetic particle-based materials and thermo-responsive polymer materials do allow highly localized surface deformations, the forces generated have been limited to the nN and pN range^32^,^35^.

Here we report a novel 2D active material platform that permits directional, remotely controlled, and highly localized surface deformation in the μN force range that can be used to micro-manipulate cells grown on the surface. This material is a new type of Hydrogel-Actuated Integrated Responsive System (HAIRS), which is designed to undergo photothermally triggered actuation of user-selected regions. HAIRS is a class of hybrid material system composed of a passive array of microstructures embedded in a stimuli-responsive hydrogel layer that converts environmental and chemical signals (for example, pH, temperature) into reversible mechanical deformations^36^,^37^,^38^,^39^. In the majority of the HAIRS systems previously reported, the microstructure actuation occurred in response to a homogenous environmental stimulus, as most of the possible stimuli are extremely difficult to pattern^37^,^38^,^39^. The only way to achieve highly localized actuation of the HAIRS was to polymerize the hydrogel in patterns^38^,^40^. Here we established a method to remotely control localized microstructure actuation with microscale spatial resolution and fast response rates by using temperature-responsive PNIPAAm hydrogel layers loaded with light-sensitive gold nanorods (AuNRs), and exercising control over the location of the microstructure actuation by patterning the light stimulus (Fig. 1). PNIPAAm hydrogels undergo a reversible and significant volume reduction above the lower critical solution temperature (LCST) of ∼32 °C, when polymer-water phase separation occurs and the gel network collapses as it expels bound water^41^. Plasmonic nanoparticles (NPs) exhibit strong light absorption in specific spectral ranges and release considerable amounts of heat due to non-radiative relaxation of excited states^42^,^43^. When the two are coupled, sample illumination through exposure to light at wavelengths that coincide with the NPs absorption maxima allows for photothermal heat generation^44^,^45^,^46^, and provides a means to locally raise the hydrogel temperature above the LCST in small, defined areas (Fig. 1). The photothermally driven volume reduction of the PNIPAAm-NP combination has proven useful for previous biomedical applications, as the relatively low LCST of PNIPAAm signifies that the operational temperature range of the system is safe, and both the polymer and NPs are known to be biocompatible^34^,^47^,^48^,^49^,^50^. On the basis of type of incorporated NP, HAIRS platform could be designed to selectively respond to light wavelengths in different regions of the spectrum^51^. We selected AuNRs because the wavelength of their absorption peak can be tuned by adjusting their size and morphology to coincide with the red to near-infrared (NIR) window (650–900 nm) that is safest for living tissue^52^,^53^.

This new HAIRS cell culture platform is similar in terms of the culture conditions (2D), the degree of cell deformation (0–45%), and the response time (0.1 Hz), to the commercial mechanical cell culture systems (for example, Flexcell International)^11^,^12^,^13^,^26^,^27^,^28^, while allowing us to apply uniaxial tension to defined portions of single cells that can be controllably oriented on the substrate at locations on the growth surface that are not predetermined during fabrication—a feature that is absent in the existing mechanical cell culture approaches. The degree of resolution and precision over the cell micro-manipulation (<10 μm) is therefore closer to what is achievable with the single cell micro-manipulation techniques^16^,^17^,^18^,^19^,^20^,^21^,^22^, but with a strain profile that better reproduces the types of forces that characterize organ and tissue distortion^13^,^26^,^27^.

## Results

### PNIPAAm-AuNR hydrogel preparation and characterization

The composite PNIPAAm-AuNR hydrogels were made by mixing various amounts of a stock solution of methoxy-polyethylene glycol (mPEG)-stabilized AuNRs dispersed in dimethyl sulfoxide (DMSO) into the hydrogel precursor solution containing *N*-isopropylacrylamide (NIPAAm), the cross-linker *N*,*N′*-methylenebisacrylamide (bis-AAm), and a ultraviolet-initiator dissolved in DMSO. The mPEG-stabilized AuNRs were synthesized according to a modified published procedure^54^,^55^. Their average length and width were 37.1±2.2 nm and 9.1±0.8 nm, respectively, giving an average aspect ratio of ∼4 (Fig. 2a inset, Supplementary Figs 1,2). These NIPAAm-AuNR precursor solutions with different AuNR concentrations were used for the preparation, via ultraviolet-triggered polymerization, of the thin hydrogel films in the hybrid HAIRS materials (abbreviated as HAIRS-*x*NR hereafter), and of the macroscopic ∼1.2 × 5 mm bulk hydrogel samples (abbreviated as HG/*x*NR hereafter) that we used for characterization, where *x* represents the vol% of the AuNR stock solution (62.2 μg ml^−1^, ∼1.5 × 10^12^ nanorods ml^−1^, see Supplementary Information) in the composite gel precursor mixture.

The HG/*x*NR samples were prepared in order to assess the effect of the PNIPAAm hydrogel on the optical properties of the AuNRs, and of the incorporated AuNRs on the gelation and physical properties of the PNIPAAm hydrogel. Ultraviolet–vis absorption measurements of the samples in the swollen state reveal successful entrapment of the AuNRs in the polymer network (Fig. 2a, Supplementary Figs 3,4). The longitudinal plasmon band appears at *λ*_max_∼815 nm, which is similar to the mPEG-stabilized AuNRs in water (Supplementary Fig. 1), and well within the NIR window. Morphological studies of the composite HG/*x*NR hydrogel using TEM (Supplementary Fig. 4) provide further confirmation regarding a uniform distribution of the AuNRs within the hydrogel matrix. Covalent attachment of the AuNRs to the polymer network was not necessary, as the AuNRs, once embedded in the crosslinked hydrogel network, cannot easily diffuse and escape from the network (Supplementary Fig. 5). Importantly, the presence of the AuNRs did not change the swelling properties of the PNIPAAm hydrogel significantly at the concentrations we tested. The swelling ratio was similar for all samples (Supplementary Table 1). These results all point to a uniform, low-density distribution of the AuNRs in the hydrogel network even at the highest concentrations used (HG/50NR).

### HAIRS-*x*NR sample preparation

The fabrication approach used to realize the hybrid polymer-nanoparticle HAIRS material is schematically shown in Fig. 2b. The NIPAAm-AuNR precursor solution was allowed to wet the microstructures and was then cured into a thin film under ultraviolet illumination (∼18 mW cm^−2^) with a coverslip as a confining upper layer. The flexible microstructure substrate consisted of rectangular arrays of 5–10 μm-long, 2 μm-wide and 12 μm-high microplates (Fig. 2c) that were fabricated using Norland Optical Adhesive 61 (NOA61) via polydimethylsiloxane double replica moulding from a silicon master (Supplementary Fig. 6), as described in previous publications (see Methods and Supplementary Information for more details)^37^,^56^.

### Photothermal properties of the HG/*x*NR hydrogel samples

The photothermal conversion of light to heat in the HG/*x*NR samples containing various concentrations of AuNRs (0–50%v/v) was visualized with a forward looking infrared (FLIR) camera (Fig. 3). The experimental set-up and procedure are described in the Methods section and Supplementary Fig. 7. Generally, a NIR (808 nm) laser beam from a laser diode (Thorlabs) was focused on the fully swollen samples, and an IR camera (FLIR ATS, France) was used to monitor the change in the hydrogel temperature over time. Significant heating of all the samples containing AuNRs was observed almost immediately after turning on the light source, while the HG/0NR sample remained close to the background temperature. The peak temperature reached and the area of the heated region were dependent on both the concentration of the AuNRs and the power of the laser (Fig. 3b,c; Supplementary Fig. 8).

### Photothermally triggered actuation of the HAIRS-*x*NR samples

The light-triggered heating and actuation of the HAIRS-*x*NR material occurs by the same localized photothermal heating mechanism that was demonstrated for the HG/*x*NR bulk samples (Supplementary Fig. 8d–h)^45^,^51^. To both actuate and image the HAIRS-*x*NR samples at a relevant scale for cell micro-manipulation experiments, and to test the resolution limit of the localized microstructure actuation, we built a custom microscope setup that focuses the NIR laser light (808 nm) down to a 10–40 μm diameter area on the sample surface at × 63 magnification and allows simultaneous sample imaging and laser exposure (see Methods). The light-to-heat conversion in the irradiated portion of HAIRS-*x*NR samples containing AuNRs produces sufficient heat to locally raise the temperature above the hydrogel LCST of 32 °C, thus causing the hydrogel to contract within seconds in a small region and actuate the neighboring microstructures, which appear as overlapping tiles when viewed from the top (Fig. 4). In comparison, exposing HAIRS-0NR samples to the NIR light does not raise the hydrogel temperature sufficiently to cause its contraction. Heating and cooling the bath to actuate the PNIPAAm gel with or without AuNRs takes several minutes at best, and results in the bending of all the microplates on the surface (Fig. 4).

Images taken from movie sequences of a series of HAIRS-*x*NR samples actuated with the same power of laser light but using increasing amounts of AuNRs show results that echo those from the illumination of the HG/*x*NR samples with NIR light (Figs 3 and 5). No actuation was observed following the localized NIR irradiation of HAIRS-0NR samples (Supplementary Fig. 8d, Movie 1), while either increasing the AuNR concentration (Fig. 5a) or the laser power (Supplementary Fig. 8e–f, Movie 2) increased the size of the actuated area.

The movement of individual microplates during the actuation cycle depends on several parameters that have an impact on the interaction of the isotropically volume-changing hydrogel with the stiff microplates. In our experiments, the region where the hydrogel contracts is roughly circular in the 2D plane of the HAIRS surface, and can be adjusted down to a ∼10 μm diameter based on the experimental parameters. On the other hand, within this circular area, the motion of the tips of the 2 × 10 μm rectangular microplates is uniaxial along the axis of their shorter dimensions where the resistance to bending is effectively lower (Fig. 5). The degree to which each microplate bends along this axis depends on its position with respect to both the outer limit of the area where the gel is contracted, and the centre region where the bending direction of the microplates reverses, which is marked by a blue dashed line in our data (Fig. 5b,c; Supplementary Fig. 9). The tips of the microplates on either side of this line shift less than the tips of the microplates nearer to the edge of the area where the gel is contracted. Eventually, the shift drops off again as less force is generated locally by the hydrogel polymer network, and therefore there is a definite boundary to the surface deformation. At higher AuNR concentrations, the size of the area over which the hydrogel contracts increases for a given laser power, and consequently the microstructures bend to a greater degree (Fig. 5d, Supplementary Figs 8 and 9b, Movies 3–5). The motion of the microplates also depends on their cross-sectional geometry (Supplementary Fig. 10, Movie 6)^37^,^38^. When microplates with a lower aspect ratio cross-section (2 × 5 μm) are actuated, there is an additional lateral component to their motion, towards the centre of the contracted area. Conversely, microplates with very large aspect ratio cross-sections (2 × 30 μm) will show increased bending of one portion of the microplates compared to the rest.

The response time of the HAIRS-*x*NR material depends on the photothermal heating, and also on the polymer microstructure material and microstructure geometry (Fig. 5, Supplementary Information)^37^,^38^. For the HAIRS-*x*NR samples with 2 × 10 μm microplates shown in Fig. 5, the heating and gel contraction occur rapidly after the laser is turned on, and the microplates are close to their peak positions after ∼3 s, and fully stabilized at ∼4 s. After the sample illumination is stopped, the heat diffuses away rapidly and the gel swells. The microplates take several seconds to relax back to a nearly upright position, although it takes several minutes for them to return to a fully upright position (Supplementary Fig. 9). As a result of the rapid response of the system to both heating and cooling processes, microplates within the irradiated region can be cyclically actuated by pulsed light exposure. Maximum cycle frequencies of close to ∼0.1 Hz can be achieved with a 5 s ‘ON’ light pulse and a 5 s ‘OFF’ recovery time for each cycle (Fig. 5c). The microplates can also be transiently actuated in complex patterns by moving the sample on a mechanical stage under continuous light exposure (Fig. 5e, Supplementary Movie 7).

Finally, our results show that the microplate behaviour is stable over many repeated actuation cycles (Fig. 5c, Supplementary Fig. 9, Movies 3,4). In addition, by testing the response of microplates in a single selected location to cyclic actuation at time points spaced eight days apart, we have ensured that the HAIRS-*x*NR samples do not age rapidly (Supplementary Fig. 11, Movie 8).

### Simulation of heat generation and diffusion

Direct measurement of the temperature in the small, cylindrical volume of the HAIRS-*x*NR sample that is exposed to the NIR laser light is difficult, although our results show that shortly after exposure, the system reaches a steady state where the size of the actuated area stops increasing, indicating that the temperature distribution is no longer changing (Supplementary Fig. 12, Movie 9). Finite element method simulations with the COMSOL 5.2 software show that the temperature at the upper interface of the PNIPAAm-AuNR hydrogel film is always lower than the temperature inside the film (Supplementary Fig. 13). When the hydrogel collapses downward as the network contracts, the temperature of the composite rises as the AuNR concentration per unit volume increases, but so does the distance between the upper boundary of the hydrogel and the basal plane of the cells. Even at the highest concentration of AuNRs that we tested (HAIRS-50NR), the calculated peak temperature and interfacial temperature do not exceed 36.5 °C and 34.4 °C, respectively (see Supplementary Information).

### Cell adhesion and growth on the HAIRS-*x*NR material

**Figure 1:**
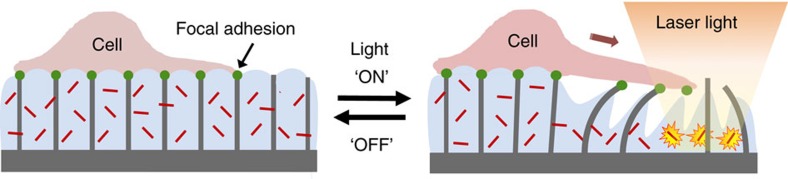
Schematic of cell manipulation by light-triggered actuation of the responsive hydrogel. At the point where the laser beam is focused, the hydrogel contracts, the microstructures bend, and the cell’s attachment points to the microstructure tips will be displaced by several microns. Note that since the cell is not attached to the contracting gel, it is positioned away from the heated gel region.

**Figure 2:**
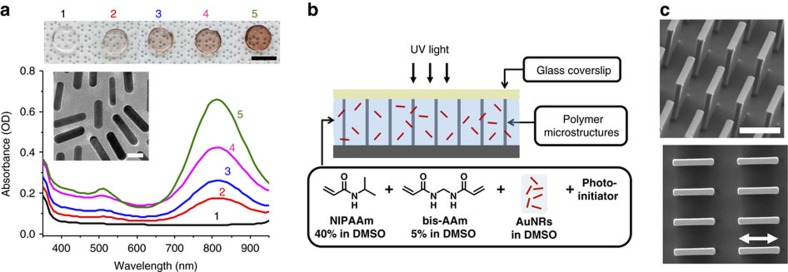
Characterization of composite HG/*x*NR samples and HAIRS-*x*NR sample preparation. (**a**) Bottom: ultraviolet–vis absorption spectra (in units of optical density (OD) of HG/*x*NR samples with various concentrations of AuNRs: (from bottom to top) 1: 0 μg ml^−1^ (HG/0NR); 2: ∼3.4 μg ml^−1^ (HG/10NR); 3: ∼4.9 μg ml^−1^ (HG/20NR); 4: ∼7.8 μg ml^−1^ (HG/30NR); 5: ∼12.2 μg ml^−1^ (HG/50NR). The inset shows a TEM image of the AuNRs in DMSO. The scale bar is 10 nm. Top: a photograph of the corresponding samples with increasing AuNR concentrations. The scale bar is 5mm. (**b**) Schematic of the fabrication procedure for the HAIRS-NR system where the AuNR-loaded hydrogel precursor solution is cured as a thin film wetting the microplate array. (**c**) 45° side-view (top) and top-view (bottom) scanning electron microscope (SEM) images of a rectangular array of 2 μm-thick, 10 μm-long and 12 μm-high polymer (NOA61) microplates used for the construction of HAIRS-NR material. The scale bar (top) and the arrow bar (bottom) are 10 μm.

The HAIRS-*x*NR materials were tested in static cell culture conditions for their ability to allow attachment and growth of cells from the D1 ORL UVA murine mesenchymal stem cell line. In addition to the increase in volume of PNIPAAm hydrogels below the LCST of 32 °C, PNIPAAm also becomes non-adhesive to cells below the phase change temperature^47^,^48^. To constrain the D1 cells into forming attachments only on the tips of the microstructures that emerge from the hydrogel layer, we chose to culture the cells on the HAIRS-*x*NR samples at 31 °C so that the PNIPAAm hydrogel was in its swollen, and hence also non-adhesive, phase, with an otherwise typical culture procedure (Figs 1, 6). Low temperature growth conditions are known to slow cell growth, which we observed with the D1 cells being cultured in standard tissue culture dishes at 31 °C (Supplementary Fig. 14), but are not known to be seriously disruptive to cells in other ways^57^,^58^,^59^.

From the confocal images of our samples, it can be seen that the cells’ positions and focal adhesions coincide with the locations of the microplate tips (Fig. 6a, Supplementary Fig. 15)^3^, and that the PNIPAAm hydrogel was in its swollen and non-adhesive state during the cell growth period (Fig. 6b). The fact that the larger areas covered by PNIPAAm in between the individual columns of microstructures and surrounding the entire array remain free of cells also supports the conclusion that the cells are forming adhesions only to the exposed microplate tips, and not to the hydrogel layer (Fig. 6, Supplementary Figs 16,17). In control experiments where we plated the cells on the microstructure substrates without the PNIPAAm hydrogel layer, the cells were able to grow over the entire surface area (Supplementary Figs 18,19).

Since the cells are only able to attach to the microplates, which are interspersed in the non-adhesive hydrogel on the HAIRS-*x*NR surface, the spatial arrangement and cross-sectional dimensions of the microplates provide the opportunity to control the cells' shapes, positions and alignment directions (Fig. 6). We explored how the different geometric parameters of the microstructure array could be used to impose a desired alignment direction on the cells with respect to the axis of the linear surface deformation by testing samples with various microplate cross-sectional geometries and spatial arrangements, with the rectangular microplates ranging in size from 2 × 5 μm to 2 × 30 μm, with inter-column separations ranging from 5 to 30 μm, and inter-row separations of 5 μm (Supplementary Fig. 20). On the 2 × 5 μm, closely spaced microplates, the cells do not show a large preference for a given alignment direction (Fig. 6c). On microplates longer than 10 μm, the cells align with the microplates (Fig. 6d) in a manner similar to the direction that has been reported for many cell types growing on micro-grooved substrates^26^,^27^,^60^,^61^. However, if the available adhesive area in that direction becomes limited by wide, non-adhesive gel regions as the microplate columns become separated by 10 μm or more, the cells switch their orientation and begin to align perpendicular to the plates, along the microplate columns (Fig. 6b,e, Supplementary Fig. 16)—a phenomenon that has not been observed in any previous studies. Very different cell alignment patterns occur when the cells are grown on microstructure samples without the PNIPAAm hydrogel (Supplementary Figs 18,19). Conversely, we observed very similar cell growth patterns on flat samples where the non-adhesive pattern created by the PNIPAAm hydrogel in the HAIRS-*x*NR surface plane was recreated with a poly(ethylene glycol) (PEG, MW∼5,000) functional group (Supplementary Fig. 21), suggesting that the cell alignment patterns that can be fine-tuned on the HAIRS-*x*NR material are the result of the spatial patterning imposed on the hydrogel by the physical presence of the microplates, which cannot be reproduced by either of the components alone. Importantly, the patterning of the available adhesive area on the sample surface combined with the low-temperature growth conditions do not seem to harm the cells, and over the course of several days in culture, the cells are able to grow and migrate (Supplementary Fig. 17).

### Cell micro-manipulation

Images from movie sequences taken during cell micro-manipulation experiments with the HAIRS-*x*NR material display the deformations of selected cells under applied mechanical stress (Fig. 7, Supplementary Fig. 22). The cell deformations are particularly visible in Supplementary Movies 10–14.

In the experiment shown (Fig. 7), the NIR laser light was focused to a region on the sample surface underneath the upper portion of the cell on the right. Within 3s from when the laser is turned on, the cell body visibly changes shape due to the movement of the microstructures beneath, and the centre-to-centre distance between the two microplates underneath the widest part of the cell body increases from 9 to 13 μm (Fig. 7a,c). This shift translates to a local degree of cell elongation close to ∼45%. The observed change of the cell's shape is due to the redistribution of the cell’s internal stresses in response to this significant externally applied local stress^32^. The images of the underlying microstructures (Fig. 7b,d) can be used to determine the exact strain profile, and show that in this experiment the strain is concentrated in the upper regions of the cell. The cell was distorted to a similar degree during four subsequent 3s laser pulses (Supplementary Movie 10), but nonetheless resumed its original shape when the microplates returned to an upright position (Fig. 7e). A cell viability assay performed two hours later did not show any signs of cell mortality (Fig. 7f). Other experiments with similarly vigorous strain conditions were performed, and the perturbed cells were observed to resume their original morphologies within a few seconds from the laser being turned off (Supplementary Fig. 23), and did not show any sign of nuclear staining during the subsequent viability assay (Fig. 7g,h, Supplementary Fig. 24). In some cases, the stimulated cells were already migrating by the time they were imaged (Fig. 7h). Finally, as a high degree of control over the applied stimulus is desirable, we further demonstrated that by changing the experimental parameters, such as the laser intensity and the AuNR concentration in the PNIPAAm hydrogel, it is possible to apply a subtler and more spatially resolved stimulus to the cells (Fig. 5a, Supplementary Fig. 22, Movies 11,12).

## Discussion

We have presented a new active cell culture material that allows highly localized, directional, and reversible deformation of the cell growth substrate, with control at scales ranging from the entire surface to the subcellular, and response times on the order of a few seconds. This material has a hybrid architecture composed of a polymeric array of microstructure actuators (a ‘skeletal’ component) embedded in a stimuli-responsive PNIPAAm hydrogel layer (a ‘muscle’), with the microstructure tips serving as anchorage points for adherent cells.

**Figure 3:**
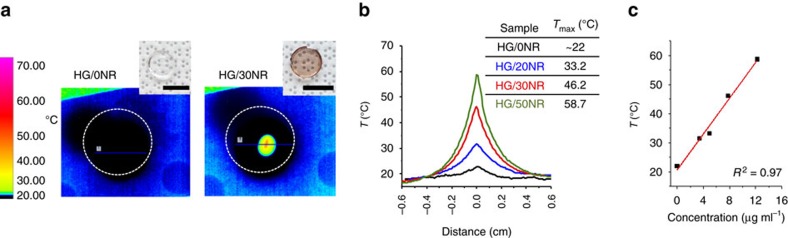
Photothermal heating of the composite HG/*x*NR. (**a**) Representative infrared images of the photothermal response in HG/*x*NR samples (digital photographs are shown in insets). The samples were irradiated at a constant power (∼85 mW). The exposure time was 5 s. The dotted circles indicate the sample boundaries. The local temperature increase is indicated by a rainbow colour scheme in the infrared images. (**b**) Temporal temperature profiles (*t*=5 s) extracted from the infrared images across the bulk HG/xNR samples with various concentrations of AuNRs. (**c**) The peak temperature increases with increasing AuNR concentration.

**Figure 4:**
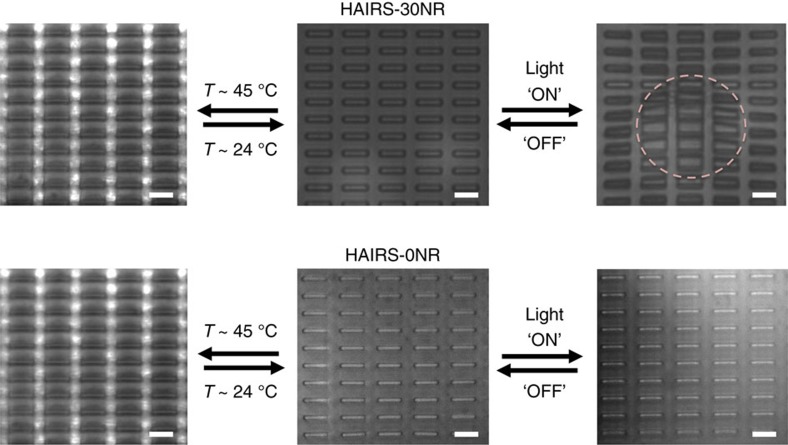
Comparison of photothermal and thermal actuation. Representative top-view images of HAIRS-*x*NR samples showing different modes of actuation. The two central images show HAIRS samples before actuation, where the central top image corresponds to a HAIRS-30NR sample and the bottom image corresponds to a HAIRS-0NR sample containing no nanorods (control). The left column represents homogeneous heating and cooling cycles of samples using a hot plate. The right column shows photothermally triggered localized heating and actuation (laser power ∼18 mW). In either experiment, the increase in temperature above the hydrogel LCST results in the contraction of the hydrogel and the bending actuation of the micro-plates, which appear as a pattern of overlapping tiles when viewed from the top. In the homogeneous heating and cooling cycles using a hot plate, all of the microplates actuate in both the HAIRS-30NR and the HAIRS-0NR samples displaying no localized effects. The NIR light exposure of the HAIRS-30NR sample produces the actuation/bending of the microplates only within the irradiated region. The pink dashed circle in the upper right image marks the area where the microplates are actuated. No actuation or bending was observed following localized NIR irradiation of HAIRS samples not containing AuNR. All scale bars are 10 μm.

**Figure 5:**
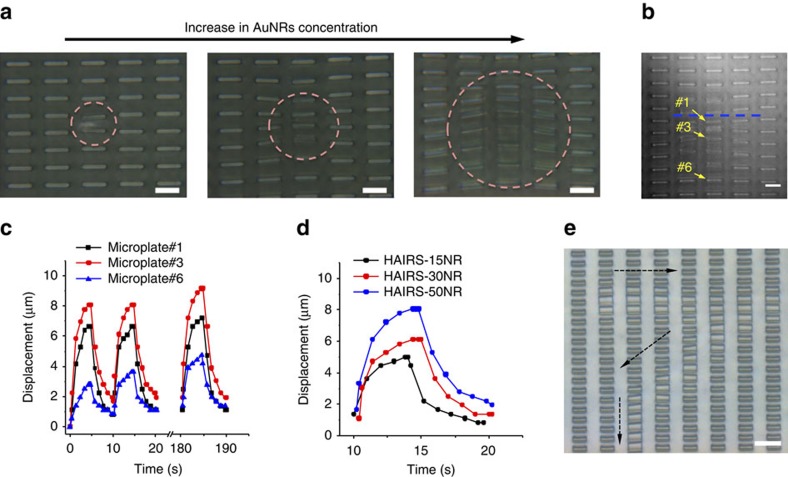
Photothermal heating of HAIRS-*x*NR samples. (**a**) Photothermal response in HAIRS-*x*NR samples with different AuNR concentrations irradiated at a constant power (∼11 mW). The pink dashed circles mark the area where the microstructures are actuated. (**b**) Sample image taken from the beginning of a movie sequence recorded during an experiment with repeated 5 s ‘ON’ (∼18 mW) and 5 s ‘OFF’ illumination cycles of a HAIRS-50NR sample (Supplementary Movie 4). The blue dashed line marks where the bending direction of the microplates reverses. The movement of microplates located at different distances from the dashed line was tracked over multiple actuation cycles in HAIRS-15NR, HAIRS-30NR and HAIRS-50NR samples to characterize their displacement profiles. (**c**) Displacement as a function of the distance from the central line where the bending direction of the microplates reverses. The movement of the microstructures #1, #3 and #6 (labelled in (**b**)) over time in a HAIRS-50NR sample (Supplementary Movie 4) is shown. (**d**) Displacement as a function of the AuNR concentration. The movement of the microstructure #3 (labelled in (**b**)) during a single actuation cycle for HAIRS-15NR, HAIRS-30NR (Supplementary Movie 3), and HAIRS-50NR (Supplementary Movie 4) samples is shown. (**e**) Image taken from a movie sequence where the HAIRS-*x*NR sample was moved as the NIR laser was kept focused on the same spot. The black arrows track the motion of the sample. All scale bars are 10 μm.

By incorporating light-sensitive AuNRs into the temperature-sensitive PNIPAAm hydrogel layer that supports the array of deformable microstructures, we found a means of remotely triggering local heating of the PNIPAAm network with focused illumination on the sample surface, thus confining the microstructure actuation to small, defined areas down to <10 μm diameter (Figs 4, 5a, Supplementary Fig. 8). We confirmed that the heating mechanism is dependent on photothermal conversion by the AuNRs, and that the irradiation of the other components in the system does not produce enough heat to raise the temperature above 32 °C (Fig. 3a,b, Fig. 4, Supplementary Fig. 8). In fact, for any given set of experimental parameters, the area over which the composite PNIPAAm-AuNR hydrogel contracts in the HAIRS-*x*NR samples shows over what distance the local temperature drops off to below the gel's transition temperature (∼10–40 μm) (Fig. 5a). Due to the highly localized volume of the heated region in our system, both the hydrogel swelling and de-swelling occur rapidly because the heat source and sink are very close to one another and therefore diffusion only has to take place over very short distances (Figs 4 and 5b,d). In comparison, heating and cooling the bath to actuate the microstructures embedded in the PNIPAAm-AuNR gel takes several minutes at best, and does not permit local actuation unless the hydrogel film itself is polymerized in patterns (Fig. 4). The result is that the photothermal actuation of PNIPAAm provides a significantly greater degree of spatiotemporal control than is possible with direct thermal actuation, thus offering a means to generate stresses at the sub-cellular scale.

For the technique to be appropriate for live mammalian cell experiments, the temperature at the boundary of the cells cannot exceed ∼39 °C. Directly measuring the temperature in such a small volume is difficult. Our results from actuating the HAIRS-*x*NR samples show that for longer laser exposure times, the system rapidly reaches an equilibrium where the size of the actuated area on the sample surface remains constant over time and the boundary of this area indicates, where the temperature decreases to below the hydrogel LCST of 32 °C (Supplementary Fig. 12, Supplementary Movie 9). To better estimate the temperature distribution in the system, we performed finite element method simulations (Supplementary Information). The results of the calculations show that even at the highest concentrations of AuNRs (HAIRS-50NR), the temperature at the upper boundary of the hydrogel does not exceed 36.5 °C (Supplementary Fig. 13). In addition, the heating in the system occurs within a few milliseconds, and therefore is not a limiting factor of the actuation speed. By copolymerizing the PNIPAAm hydrogel with hydrophobic or hydrophilic co-monomers or by performing end-group transformations, the transition temperature of the system can readily be shifted to closer to physiological temperatures^62^, although care would have to be taken during cell manipulation experiments to avoid over-shooting the temperature range above which cells begin to experience heat shock damage. For example, replacing a fraction of the *N*-isopropylacrylamide (NIPAAm) monomers with *N*-isopropylmethacrylamide (NIPMAAm) will shift the LCST to ∼38 °C (ref. 45). Thus, by fine-tuning the hydrogel composition and the laser exposure parameters, it should be possible to raise the transition temperature of the system (and the cell culture temperature) and actuate the microstructures without exceeding 37 °C at the basal plane of the cells.

Although, the PNIPAAm-AuNR composite hydrogel is responsible for the microstructure actuation, the microstructure material and 3D geometry play a very important role in the timing and direction of the actuation. We used NOA61, which has an elastic modulus of 1.0 GPa, and were able to achieve cycling frequencies on the order of 0.1 Hz. Keeping all other experimental parameters constant, one can achieve faster cycling rates with a material with a lower elastic modulus^37^,^38^. Another method to increase the cycling rate would be to use narrower microstructures (<2 μm), as the microstructure geometry also impacts the amount of force required to deflect the tip in a specific direction (Supplementary Information)^37^,^38^. For a rectangular microplate with dimensions of *h*=12 μm, *w*=2 μm and *l*=10 μm, the magnitude of force required to bend and displace the tip by 8 μm can be estimated to be ∼100 μN (Fig. 5c), which also provides an estimate of how much force is transmitted to a cell via the displacement of the focal adhesions with the microplate. We specifically chose to make our HAIRS-*x*NR samples with the rectangular microplates in order to ensure that the microstructure displacement would be uniaxial, along the axis of their shortest dimension where the resistance to bending is effectively the lowest (Fig. 5a,b). Uniaxial strain is a highly relevant strain profile for studies in cell mechanotransduction^13^,^26^,^27^, and the ability to uniaxially and repeatedly displace some points on the surface at user-defined locations and times is not possible with other active cell culture materials^30^,^31^,^32^,^33^,^34^. Flexible membrane devices such as the Flexercell can be operated at higher frequencies (up to 10 Hz), but there is no option of only activating a small area on the membrane^11^,^12^,^13^,^26^,^27^,^28^. Thus, the optically induced local heating mechanism, paired with the contraction of the AuNR-loaded hydrogel and the associated actuation of the patterned array of microstructures, enables remote and precise spatiotemporal control over reversible, repeatable, rapid and directionally defined surface deformations.

**Figure 6:**
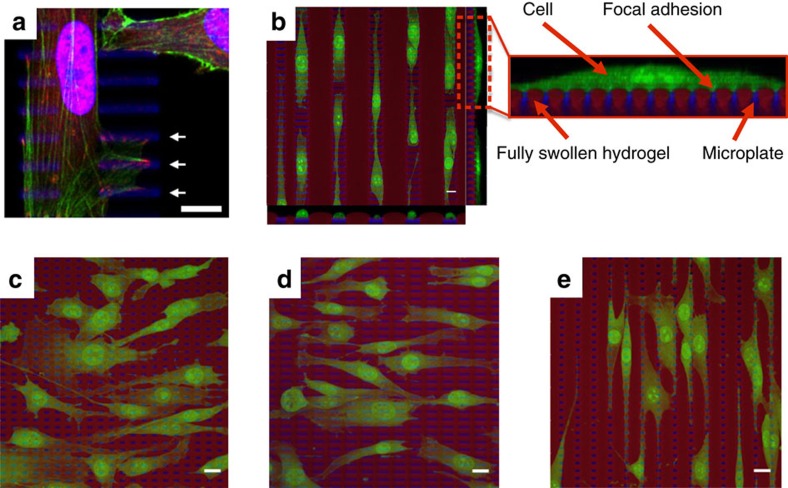
Cell growth on the HAIRS-*x*NR material. (**a**) The cells form focal adhesions on the tips of the microstructures. Representative reconstructed 3D confocal image showing a cell that was immunostained for the focal adhesion protein vinculin (green: actin (phalloidin fluorescent counterstain), red: fluorescently labelled anti-vinculin antibody, blue: nucleus (DAPI fluorescent counterstain) and autofluorescence from the microstructures). The white arrows point at different microplates where multiple micron-scale vinculin-positive complexes are evident. The microstructure dimensions and spacing are 2 μm wide, 10–15 μm long, 5 μm between neighboring rows and 10–25 μm between neighboring columns. (**b**–**e**) Representative 3D confocal images showing examples of the cell growth and alignment after 24 h at 31 °C on substrates with different microstructure geometries. The cells (green) were grown on top of fluorescently labelled HAIRS samples (blue: microstructures labelled with pyrromethene 546, red: hydrogel in its swollen state labelled with DY-485 XL, green: cells labelled with HCS CellMask Deep Red Stain). The microstructure dimensions and spacing in the surface plane are: (**b**) 2 μm wide, 15 μm long, 5 μm between rows and 25 μm between columns, (**c**) 2 μm wide, 5 μm long, 5 μm between rows and 5 μm between columns, (**d**) 2 μm wide, 10 μm long, 5 μm between rows and 5 μm between columns, and (**e**) 2 μm wide, 5 μm long, 5 μm between rows and 10 μm between columns. All scale bars are 10 μm.

**Figure 7:**
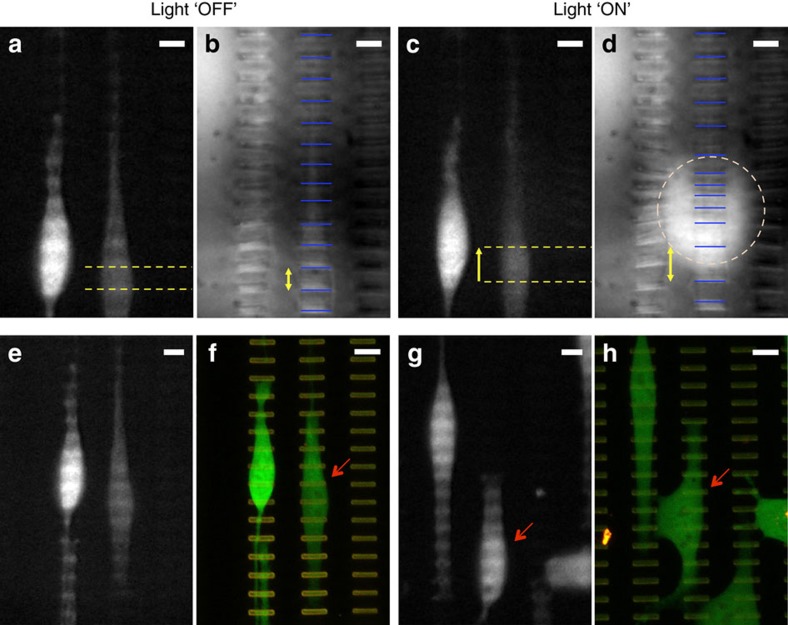
Cell micro-manipulation with targeted actuation of the HAIRS-*x*NR material. (**a**–**e**) Images taken from a movie sequence (Supplementary Movie 10) recorded during a cell micro-manipulation experiment showing epifluorescence images of the cells on the left and reflected brightfield images of the underlying microstructures on the right. The cells were cultured on the HAIRS-30NR sample for 24 h before the experiment. The laser is focused at the centre of the pink dashed circle that outlines the area where the hydrogel is contracted and the microplates are actuated. The cells are labelled with the fluorescent CellTracker Green CMFDA dye. Images of two cells before the start of the experiment (**a**) and of the same cells ∼3 s after the laser (∼18 mW) is initially turned on (**c**) are shown. The cell on the right undergoes a significant change in shape due to the movement of the microstructures underneath it (**b**,**d**). The locations of the two microstructures at both time points are marked by the yellow dashed lines, and the elongation from 9 μm (**b**) to 13 μm (**d**) of the portion of the cell overlying that region is indicated by the yellow arrows. The blue bars in the brightfield images mark the locations of the microstructure tips, and highlight the change in the distance separating the tips of adjacent microstructures after the hydrogel contraction is triggered. (**e**) Epifluorescence and (**f**) confocal images of the cells immediately after (**e**) and 2 h after (**f**) the manipulation experiment (2 × 3 s pulses at ∼18 mW, 1 × 3 s pulse at ∼11 mW, and 2 × 3 s pulses at ∼4 mW). (**g**) Epifluorescence and (**h**) confocal images of two cells before (**g**) and 2 h after (**h**) a second manipulation experiment (Supplementary Movie 13, fifteen short pulses followed by 5 min of continuous strain at ∼4 mW). Before taking the images shown in (**f**,**h**), the cells were stained with Sytox Orange Nucleic Acid Stain as a viability assay. The red arrows point to the cell that was subjected to mechanical perturbations. All scale bars are 10 μm.

The non-adhesive character of the PNIPAAm hydrogel at the temperatures below 32 °C at which the cells are cultured^47^,^48^, coupled with the geometry and placement of the microstructures in the hydrogel film, bring about several noteworthy advantages of this new active cell culture platform, as the cells are constrained into forming adhesions only to the microstructure tips, and not to the hydrogel (Fig. 6; Supplementary Fig. 15). First, the cells' contact with the heated hydrogel during the photothermally triggered contraction step is minimized (Fig. 1), which prevents a potential loss in cell viability (Fig. 7e-h). Second, the cells are subjected primarily to the directional stresses defined by the bending motions of the microstructures. Third, by designing the spatial arrangement of the microstructures in the hydrogel in such a way that the adhesive area available to the cells is confined in one or more directions, the cultured cells can be induced to align in any orientation with respect to each other and to bending axis of the microplates (Fig. 6, Supplementary Figs 16,20), and therefore relative to the direction of the applied stress (Figs 4 and 6). Thus, by causing the cells to grow in alignment with the columns of microstructures, we were able to strain the cells uniaxially along their long axis (Fig. 7). This is a highly relevant biological strain direction that can be difficult to achieve with other approaches, for example, the necessity of using micro-grooved flexible membranes that maintain the cells in alignment with the strain direction, but is a crucially important factor in determining experimental outcomes (Supplementary Movie 10–14)^13^,^26^,^27^. By extension, the geometry and spatial organization of the microstructures and the hydrogel film can also be designed to affect the cell growth patterns and the surface deformation profiles in a number of different ways, which offers many additional degrees of freedom to tailor the system towards a specific cell application.

We presented here some proof-of-concept results that highlight the potential of the HAIRS-*x*NR as a versatile platform for cell mechanical manipulation experiments. With the rectangular microplate arrays that we tested, the HAIRS-*x*NR surface deformations can be used to apply uniaxial external stress on the selected cells, which can either be maintained continuously, or applied cyclically with frequencies on the order of 0.1 Hz, and with the possibility of achieving significant cell strain magnitudes (Fig. 7, Supplementary Fig. 22, Movies 10–14). We were able to reach up to 45% local strain in portions of selected cells, while leaving the other parts of the cells undisturbed (Fig. 7a,c, Supplementary Movie 10), and our data show that, subject to the experimental parameters, the forces generated by the hydrogel contraction can easily deform cells to greater or lesser degrees (Supplementary Movies 10–14, Supplementary Fig. 22). The fact that the morphology of the selected cells after the material returns to its relaxed state is not discernably different from their original morphologies is noteworthy (Fig. 7, Supplementary Fig. 23). Seemingly, while the externally applied stress causes a redistribution of the cells' internal stresses, the equilibrium states in the absence of the applied stress were not greatly affected by the strain profiles that we tested. In addition to the cell viability assay that we performed (Fig. 7, Supplementary Fig. 24), which did not show any signs of mortality in the cells subjected to repeated cycles of applied stress that resulted in significant transient deformations, these last observations support the encouraging hypothesis that our new method makes it possible to significantly strain the cell's cytoskeletal network without causing damage. With these results, we have therefore demonstrated that this new technique combines the necessary spatial resolution, precision, and non-invasiveness to controllably probe how cells respond to localized stresses by displacing an isolated subset of a cell’s focal adhesions by a few microns along a single axis on the 2D growth surface. To our knowledge, this is not otherwise feasible.

The new HAIRS-*x*NR platform is therefore highly competitive with respect to other available cell manipulation techniques in terms of the degree of cell strain, the amount of user control over the strain conditions, its minimal invasiveness, and its versatility to being customized towards different types of cell strain experiments^11^,^12^,^63^. The degree of spatial resolution over the cell manipulation is close to what is achievable with the single cell manipulation techniques^11^,^12^,^16^,^17^,^18^,^19^,^20^,^21^,^22^, but the applied mechanical stimulus with the HAIRS-*x*NR material is a better reproduction of the types of strain that characterize organ and tissue distortion^13^,^26^,^27^,^29^. Like the flexible membrane devices such as the Flexercell, the mechanical stimulus involves the directional displacement of the focal adhesions from which the cells establish the mechanical equilibrium of their internal scaffold, with the possibility of reaching local degrees of strain that are within the physiological range and well above the threshold required for eliciting responses in cells^11^,^12^,^13^,^20^,^26^,^27^,^28^. However, with the HAIRS-*x*NR material this type of stimulus can be limited down to a fraction of a single cell, which facilitates the study of how these types of forces propagate inside of cells and cause changes^16^. No other active material enables the directional distortion of selected cells at such a small scale and with such high degrees of cell strain^30^,^31^,^32^,^33^,^34^.

In addition, the use of NIR light to trigger the transition of the PNIPAAm-AuNR composite hydrogel opens up many options for patterning and scaling the surface activation in future experiments (Fig. 5), including the generation of complex stress profiles over large areas of the surface, and also the selection of specific groups of cells within the population on the growth surface for different mechanical stimulation programs (Supplementary Movies 3–5). The types of whole cell strain profiles that would be possible with this new platform would provide an interesting contrast to the uniaxial, biaxial or equibiaxial increase in area strain patterns that are the current standard for 2D cell strain platforms^12^,^13^,^26^,^27^,^28^. According to the experimental design and the choice of strain conditions, the cells could be subjected to changes in tension along some actin stress fibres and not others, or even significant changes to their cytoskeletal organization, while their contact area with the surface remains close to constant. These new types of cell strain profiles may prove to be useful models of mechanical cues that are currently difficult to emulate *in vitro*, including the stress redistributions in cell sheets following the thinning of the basement membrane that characterize the morphogenesis of epithelial tissues^5^,^6^,^7^,^64^,^65^.

To conclude, we believe this new cell culture material could be a broadly applicable platform for different types of research that would benefit from the ability to deform the cell growth surface in a highly controlled manner. It opens up many heretofore out-of-reach possibilities for directly studying how forces propagate inside of single cells and populations of cells, and how the cells and networks integrate mechanical signals across a hierarchy of scales. This information will help to build and improve mathematical models that can predict cell behaviour^12^,^14^,^19^, and will in turn provide fundamental insights into the physical mechanisms involved in important processes such as organ development and cancer progression^4^,^5^,^6^,^7^,^8^,^9^. In addition, the HAIRS-*x*NR material’s simple hybrid structure and fabrication may enable its integration as a modular component in more complex microfluidic devices, in both static and flow configurations, which is desirable for applications in tissue engineering^29^,^66^. This unique and extremely useful set of capabilities is not matched by any other method, and thus this versatile and highly customizable material has the potential to bridge the performance gap between the existing single cell micro-manipulation techniques and the 2D cell sheet mechanical stimulation techniques, which has previously hampered research in the fields related to cell mechanotransduction^11^,^12^.

## Methods

### Materials

Gold(III) chloride solution (30 wt% in diluted HCl, 99.99%), silver nitrate (99.0%), hexadecyltrimethylammoniumbromide (CTAB, 98%), sodium borohydride (99%), L-ascorbic acid, DMSO, *N*-isopropylacrylamide (NIPAAm), 2-hydroxy-2-methyl-1-phenyl-1-propanone (DAROCUR 1173, photoinitiator for temperature-responsive hydrogel), N,N′-methylenebisacrylamide (bis-AAm, cross-linker) and fibronectin from human plasma (0.1% solution) were purchased from Sigma-Aldrich. Methoxy-poly(ethylene glycol)-thiol (mPEG-SH, MW∼5,000) was purchased from Laysan Bio, Inc. Pyrromethene 546 was purchased from Exciton. DY-485XL was purchased from Dyomics GmbH. All the chemicals, except NIPAAm (which was recrystallized from hexane), were used as received. HCS CellMask Deep Red Stain, CellTracker Green CMFDA, Live Cell Imaging Solution, and Sytox Orange Nucleic Acid Stain, Alexa Fluor 546 phalloidin, goat anti-rabbit secondary antibody Alexa Fluor 633 conjugate, DAPI nucleic acid stain (4′,6-Diamidino-2-Phenylindole, Dilactate), and 10% normal goat serum were purchased from Life Technologies and used according to the provided procedures. The anti-vinculin primary antibody (ab73412) was purchased from Abcam. Bovine serum albumin was purchased from Jackson ImmunoResearch Laboratories, Inc. Paraformaldehyde was purchased from VWR. D1 ORL UVA cells (CRL-12424) were purchased from ATCC. Norland Optical Adhesive (NOA61) was purchased from Norland products (Cranbury, NJ). Polydimethylsiloxane (Dow-Sylgard 184) was purchased from Ellsworth (Germantown, WI). Deionized water (18 MΩ) was used in all the experiments.

### Synthesis and characterization of the AuNR-mPEG

The synthesis method was adapted from a previously published seed-mediated growth method developed by El-Sayed (see Supplementary Information for details)^54^. The absorbance of the AuNRs in solution was characterized by ultraviolet–vis measurements using a Cary60 spectrometer (Agilent). Transmission Electron Microscopy (TEM) images were acquired using a JEOL 2100 microscope (Japan) with an operating voltage of 200 kV. The samples for TEM images were made by placing a drop of the AuNR solution on a TEM carbon-coated grid. The high resolution images were acquired using a Gatan Osiris digital camera. Gold ion concentrations were determined using an inductively coupled plasma atomic emission spectrometer (Massachusetts Materials Research Inc.). The concentration of the AuNR-mPEG DMSO stock solution was calculated to be 2.18 nM based on inductively coupled plasma analysis (62.2 μg ml^−1^, ∼1.5 × 10^12^ nanorods ml^−1^, see Supplementary Information).

### Preparation of the NIPAAm-AuNR hydrogel precursor solution

The composite NIPAAm-AuNR precursor solution was made by mixing various amounts (10–50%v/v) of the stock solution of polyethylene glycol (PEG) stabilized AuNRs dispersed in DMSO into the freshly prepared hydrogel precursor solution containing NIPAAm, (19–23%w/w), the cross-linker bis-AAm (0.96–1.2%w/w), and the ultraviolet-initiator 2-hydroxy-2-methylpropiophenone (DAROCUR 1173, 0.2–0.3%w/w) dissolved in DMSO. The absorbance of the HG/*x*NR samples was measured on a Benchmark Bio-Rad microplate reader in the 350–1000, nm range using the Microplate manager 4.0 software. The AuNR morphology and distribution was characterized by TEM using resin-infiltrated and cut samples (Supplementary Information).

### HAIRS-*x*NR fabrication procedure

To embed the microstructures in the light-responsive hydrogel film, a drop (∼1.2 μl cm^−2^) of the NIPAAm-AuNR precursor solution (DMSO) was placed on the surface of the microplate array and carefully spread by sandwiching the precursor solution between the patterned surface and a glass coverslip. Then, the samples were cured under ultraviolet illumination (∼18 mW cm^−2^) for 90 s. The cover slip was removed after a 30 s cooling period, and all samples were soaked in deionized water overnight to exchange the DMSO for water, and remove traces of unreacted monomer and ultraviolet initiator. Finally, the samples were incubated in a 50 °C oven for 30 min in order to test their ability to fully actuate, and also to pre-stress the microstructures.

### HG/*x*NR sample NIR irradiation and imaging procedure

The experimental set-up is described in Supplementary Fig. 7. An NIR (808 nm) laser beam from a laser diode (Thorlabs) was focused on the centre of the fully swollen hydrogel samples (wiped clean of excess water) and an IR camera (FLIR ATS, France) placed directly above the sample was used to monitor the change in the hydrogel temperature over time.

### Microscope setup used for sample actuation and imaging

Light from an 808 nm laser diode was coupled into the microscope optical train (Olympus BXFM) through a customized port and then directed and focused onto the sample surface along with the imaging light with a customized multi-bandpass filter cube and a × 63 water immersion dipping objective (Zeiss W-Plan Apochromat × 63/1.0). An additional filter placed before the camera port protected the camera sensors (Hamamatsu EM-CCD) from the laser light and allowed simultaneous sample illumination with the NIR light and low-signal level sample imaging. In a typical actuation experiment, a HAIRS-*x*NR sample was placed in a Petri dish filled with water or an aqueous buffer and mounted on the stage of the set-up. In this experimental configuration, the laser light could be focused down to a 10–40 μm diameter area on the sample surface.

### Sample preparation for cell culture

The HAIRS-*x*NR samples for the cell experiments had a hydrogel composition of 23%w/w NIPAAm, 1.2%w/w bis-AAm, 0.3%w/w DAROCUR 1173 and 15–30%v/v of the AuNR in DMSO. To prepare the HAIRS-*x*NR samples for cell culture, the samples were first dehydrated by allowing the water to be exchanged with ethanol over several steps, and were then allowed to air dry. A 10 min O_2_ plasma cleaning step (20 W, 0.3 bar O_2_) removed small amounts of hydrogel that may have submerged the tips of the microstructures during the hydrogel curing step. The samples were then rehydrated by first immersing them in ethanol, and then transferring them back to deionized water. Incubating the samples in a fibronectin solution (0.0025% in water) for 1–4 h also aided subsequent cell attachment, spreading and growth. Before plating the D1 ORL UVA cells (cell line purchased from ATCC, not tested for mycoplasma, not on ICLAC/NCBI misidentified cell lists), the samples were rinsed 3 × in water, sterilized for 30 min in 70% ethanol, and rinsed 3 × in sterile HBSS, always at room temperature. We incubated our cells just below the hydrogel LCST at 31 °C, so that the cells would only form attachments to the microstructures.

### Cell micro-manipulation procedure

The cells were fluorescently labelled with CellTracker Green CMFDA several hours before the experiment. During the experiment, the sample was transferred to a Petri dish filled with room temperature Live Cell Imaging Solution and mounted on the stage of the set-up. The sample was first scanned in the fluorescent imaging mode until a cell was selected, then the microstructure actuation was tested nearby in the reflected brightfield imaging mode. Finally, the cell was positioned at the desired location relative to the actuation position, and the NIR laser light was turned on while the sample was imaged fluorescently at a frame rate of 5 frames per s. The sample was then returned to the 30 °C incubator in full medium for 100 min, and stained with a 2.5 μM Sytox Orange Nucleic Acid Stain solution for 10 min. Finally, the live cells were imaged with a confocal microscope.

### Data availability

The data that support the findings of this study are available within the article (and its Supplementary Information files) and from the corresponding author on reasonable request.

## Additional information

**How to cite this article**: Sutton, A. *et al*. Photothermally triggered actuation of hybrid materials as a new platform for *in vitro* cell manipulation. *Nat. Commun*. **8**, 14700 doi: 10.1038/ncomms14700 (2017).

**Publisher's note**: Springer Nature remains neutral with regard to jurisdictional claims in published maps and institutional affiliations.

## Supplementary Material

Supplementary Information Supplementary Figures, Supplementary Tables, Supplementary Notes, Supplementary Methods and Supplementary References.

Supplementary Movie 1 Movie recorded during the photothermal response in HAIRS-0NR sample (control). The sample was irradiated with laser power ~68mW (1.7A). The movie is running at 2x speed

Supplementary Movie 2 Movie recorded during the photothermal response in HAIRS-50NR sample. The sample was irradiated with short laser pulses (~5s) with increasing power from ~1mW (1.0A) to ~68mW (1.7A, 0.1A increment). The movie is running at 2x speed.

Supplementary Movie 3 Movie recorded in the reflected brightfield imaging channel during an actuation cycling experiment with a HAIRS-30NR sample running at 8x speed. Data from this experiment is used for Figure 3c. The laser power is set to ~18mW (1.3A) and is turned on for ~5 s, then off for ~5s. The hydrogel composition is 19%w/w NIPAAm, 0.96%w/w bisAam, 0.2%w/w DAROCUR® 1173, and 30%v/v AuNR.

Supplementary Movie 4 Movie recorded in the reflected brightfield imaging channel during an actuation cycling experiment with a HAIRS-50NR sample running at 8x speed. Data from this experiment is used for Figure 3c. The laser power is set to ~18mW (1.3A) and is turned on for ~5s, then off for ~5s. The hydrogel composition is 19%w/w NIPAAm, 0.96%w/w bisAam, 0.2%w/w DAROCUR® 1173, and 50%v/v AuNR.

Supplementary Movie 5 Segment of a movie recorded in the reflected brightfield imaging channel during an actuation cycling experiment with a HAIRS-30NR sample. Data from this experiment is used for Supplementary Figure 8c. The laser power is set to ~4mW (1.1A) and is turned on for ~5s, then off for ~5s. The hydrogel composition is 19%w/w NIPAAm, 0.96%w/w bis-Aam, 0.2%w/w DAROCUR® 1173, and 30%v/v AuNR.

Supplementary Movie 6 Movie recorded in the reflected brightfield imaging channel during an actuation cycling experiment with a HAIRS-30NR sample at different locations on a sample running at 2x speed. Data from this experiment is used for Supplementary Figure 10a,d,c,f. (a) 2x5μm and (b) 2x30μm rectangular microplate cross-sections, respectively. While the 2x30μm microplates bend uniaxially along the axis of their shorter dimension, the 2x5μm microplates also bend along the axis of their longer dimension. The scale bars are 10μm.

Supplementary Movie 7 Movie recorded during the photothermal response in HAIRS-30NR sample (20x objective). The sample was first irradiated with short laser pulses (~5s) with increasing power from ~18mW (1.2A) to ~120mW (2.1A, 0.1A increment), then a stage with the sample was moved under continuous light exposure (~40mW, 1.4A). The hydrogel composition is 20%w/w NIPAAm, 1%w/w bis-Aam, 0.2%w/w DAROCUR® 1173, and 30%v/v AuNR.

Supplementary Movie 8 (a) Movie recorded in the reflected brightfield imaging channel during an actuation cycling experiment with a HAIRS-15NR sample running at 2x speed. The laser power is set to ~18mW (1.3A) and is turned on for ~5s, then off for ~5s. The hydrogel composition is 19%w/w NIPAAm, 0.96%w/w bis-Aam, 0.2%w/w DAROCUR® 1173, and 15%v/v AuNR. (b) The same sample was irradiated at the same spot after 8 day using same experimental conditions. The scale bars are 10μm.

Supplementary Movie 9 Movie recorded in the reflected brightfield imaging channel during an actuation cycling experiment with a HAIRS-15NR sample running at 8x speed. The laser power is set to ~68mW (1.75A) and is turned on continuously for ~2.5min. The hydrogel composition is 19%w/w NIPAAm, 0.96%w/w bis-Aam, 0.2%w/w DAROCUR® 1173, and 15%v/v AuNR. The scale bars are 10μm.

Supplementary Movie 10 Movie recorded during a cell micro-manipulation experiment with very vigorous strain conditions, running at 4x speed. Data from this experiment is shown in Figure 7, and Supplementary Fig. 23a-b. The cells and underlying microstructures are imaged in the same location with two different imaging channels: the epifluorescence imaging mode (cells, labeled with CellTracker Green CMFDA), and the reflected brightfield mode (microstructures). The laser power was set to ~18mW (1.3A) for 2x 3s pulses, then ~11mW (1.2A) for 1x 3s pulse, and finally ~4mW (1.1A) for 2x 3s pulses. The hydrogel composition is 19%w/w NIPAAm, 0.96%w/w bis-Aam, 0.2%w/w DAROCUR® 1173, and 30%v/v AuNR. The cells are labeled with CellTracker Green CMFDA

Supplementary Movie 11 Segment of a movie recorded during a cell micro-manipulation experiment running at 4x speed. Data from this experiment is shown in Supplementary Figures 22 and 23g-h. The cells and underlying microstructures are imaged in the same location with two different imaging channels: the epifluorescence imaging mode (cells, labeled with CellTracker Green CMFDA), and the reflected brightfield mode (microstructures). The laser power is set to ~4mW (1.1A), and the cell strain is maintained continuously after it is first applied. The cell reaches its fully deformed shape ~4s after the laser is turned on. The hydrogel composition is 23%w/w NIPAAm, 1.2%w/w bis-Aam, 0.2%w/w DAROCUR® 1173, and 15%v/v AuNR. The cells are labeled with CellTracker Green CMFDA.

Supplementary Movie 12 Segment of a movie recorded during a fine control cell micro-manipulation experiment running at 4x speed. The cells and underlying microstructures are imaged in the same location with two different imaging channels: the epifluorescence imaging mode (cells, labeled with CellTracker Green CMFDA), and the reflected brightfield mode (microstructures). The laser power is set to ~4mW (1.1A), and in the segment shown, the cell strain is maintained continuously after it is applied. The hydrogel composition is 23%w/w NIPAAm, 1.2%w/w bis-Aam, 0.2%w/w DAROCUR® 1173, and 30%v/v AuNR. The cells are labeled with CellTracker Green CMFDA.

Supplementary Movie 13 Segment of a movie recorded during a cell micro-manipulation experiment running at 4x speed. Data from this experiment is shown in Figure 7g, and Supplementary Figure 23c-d. The cells and underlying microstructures are imaged in the same location with two different imaging channels: the epifluorescence imaging mode (cells, labeled with CellTracker Green CMFDA), and the reflected brightfield mode (microstructures). In the segment shown, the laser power is pulsed at ~4mW (1.1A). The entire experiment is composed of 15 short pulses and 5min of continuous strain. The hydrogel composition is 19%w/w NIPAAm, 0.96%w/w bis-Aam, 0.2%w/w DAROCUR® 1173, and 30%v/v AuNR. The cells are labeled with CellTracker Green CMFDA.

Supplementary Movie 14 Movie recorded during a cell micro-manipulation experiment running at 4x speed. Two segments from the beginning and end of the experiment were combined to make the final version of the movie shorter. Data from this experiment is shown in Supplementary Figures 23e-f and 24. The cells and underlying microstructures are imaged in the same location with two different imaging channels: the epifluorescence imaging mode (cells, labeled with CellTracker Green CMFDA), and the reflected brightfield mode (microstructures). In the segment shown, the laser power is pulsed at ~4mW (1.1A). Over the entire experiment, the laser was pulsed on for 3s followed by 3s relaxation periods, for a total duration of 6min. The hydrogel composition is 19%w/w NIPAAm, 0.96%w/w bis-Aam, 0.2%w/w DAROCUR® 1173, and 30%v/v AuNR. The cells are labeled with CellTracker Green CMFDA.
