# Corrigendum: Photothermally triggered actuation of hybrid materials as a new platform for *in vitro* cell manipulation

**DOI:** 10.1038/ncomms15446

**Published:** 2017-08-14

**Authors:** Amy Sutton, Tanya Shirman, Jaakko V.I. Timonen, Grant T England, Philseok Kim, Mathias Kolle, Thomas Ferrante, Lauren D Zarzar, Elizabeth Strong, Joanna Aizenberg

Nature Communications 8 Article: 14700 ; DOI: 10.1038/ncomms14700 (2017); Published 03 13 2017; Updated 08 14 2017

The financial support for this Article was not properly acknowledged. The acknowledgements should include a reference to support, ‘by the ONR under award number N00014-16-1-3169 (Designing a Dynamic Platform that Provides Multiple Defense Mechanisms against Fouling)’ instead of ‘ONR under award number N00014-15-1-2157 (cell manipulation)’.

