# Supplementary material for: Photothermally triggered actuation of hybrid materials as a new platform for in vitro cell manipulation
Source: Nat Commun. 2017 Mar 13;8:14700. doi: 10.1038/ncomms14700 (PMC5355809; doi:10.1038/ncomms14700)
Supplement: Supplementary Information — Supplementary Figures, Supplementary Tables, Supplementary Notes, Supplementary Methods and Supplementary References. [file ncomms14700-s1.pdf]

Supplementary Figures

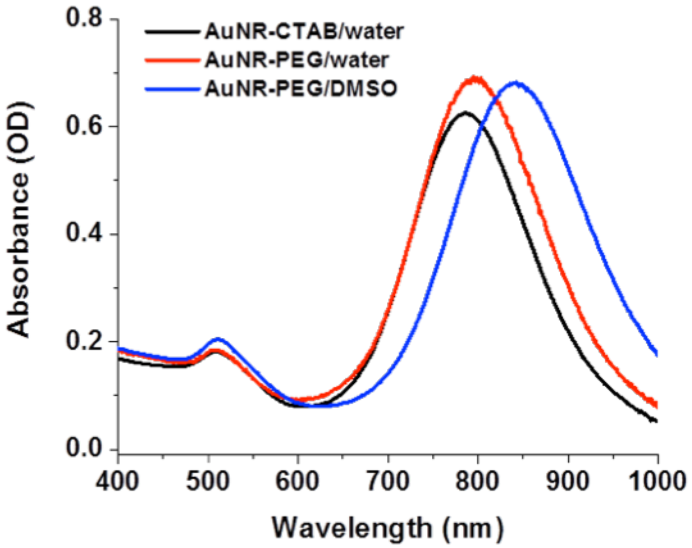

Supplementary Figure 1. UV-vis absorption spectra of AuNRs capped with CTAB (AuNR-CTAB) in water (black trace); AuNR-PEG in water (red trace); and AuNR-PEG in DMSO (blue trace).

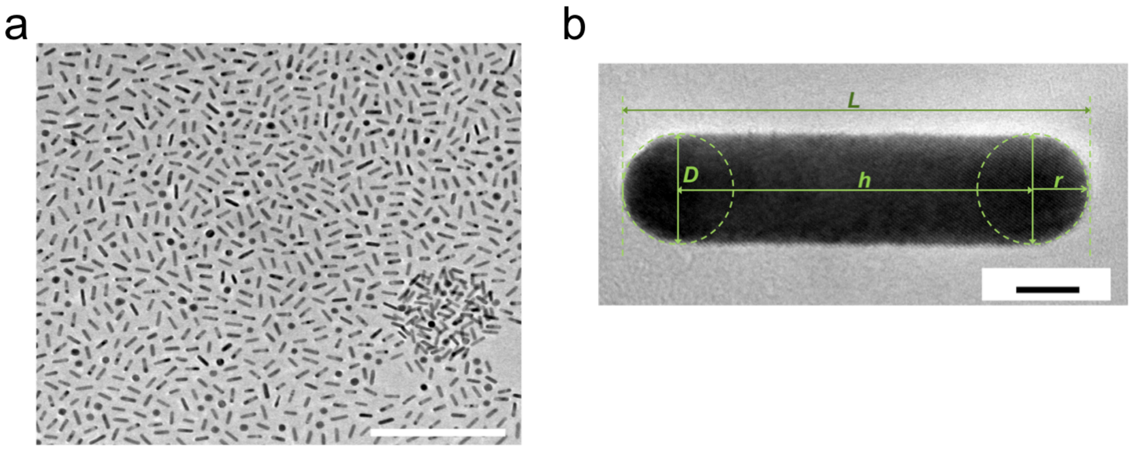

Supplementary Figure 2. (a) Representative TEM image of AuNR-PEG fraction in DMSO used for embedding in hydrogel. **(b)** High resolution TEM image of a single AuNR-PEG. The scale bars are (a) 200nm, (b) 5nm.

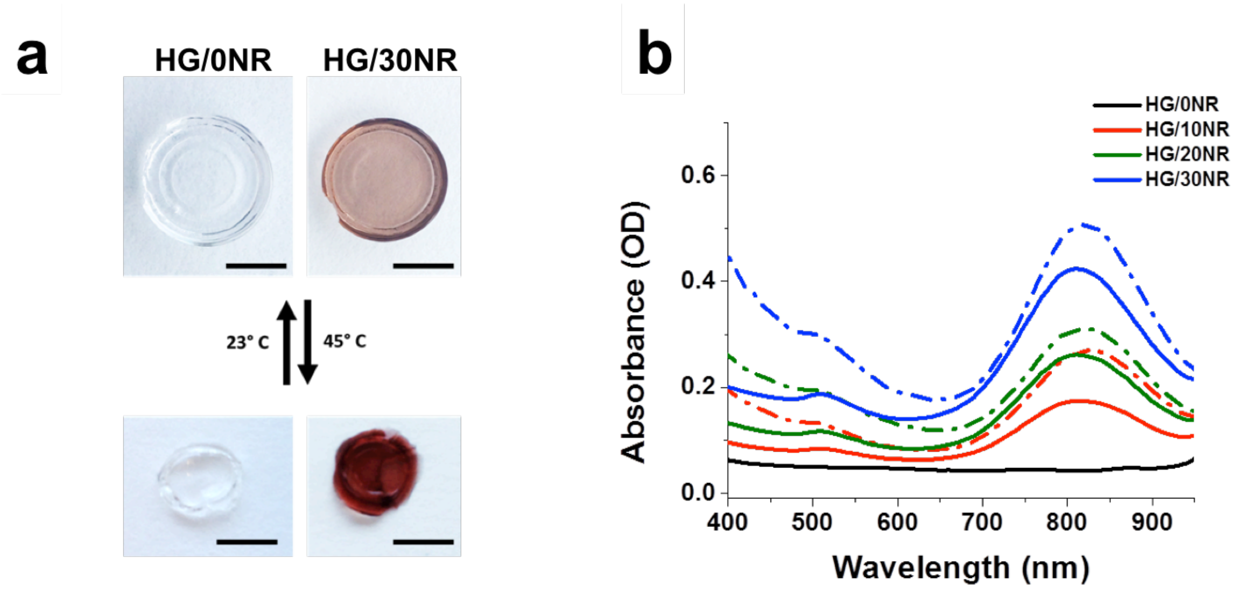

Supplementary Figure 3. Contraction/swelling of HG/*x*NR bulk hydrogel samples upon heating and cooling. **(a)** Digital photos of the HG/30NR (~7.8μg ml^{-1}) composite hydrogel samples at 23°C and 45°C. Scale bars are 0.5 cm. **(b)** Absorption spectra of HG/*x*NR at room temperature (solid lines) and at 45°C (dotted lines).

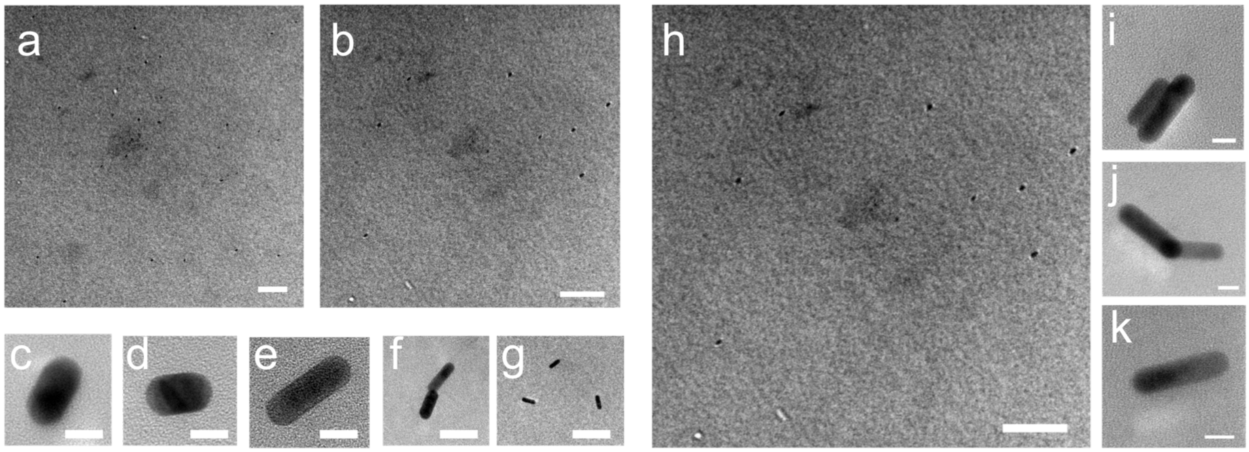

Supplementary Figure 4. Representative TEM images of PNIPAAm-AuNR bulk samples (HG/50NR). The bulk samples were infiltrated with resin, cured and cut into thin sections (100-200nm) using a microtome. The scale bars are (a, b) 0.5μm, (c, d, e) 5nm, (f, i-k) 10nm, (g) 20nm, (h) 0.2μm.

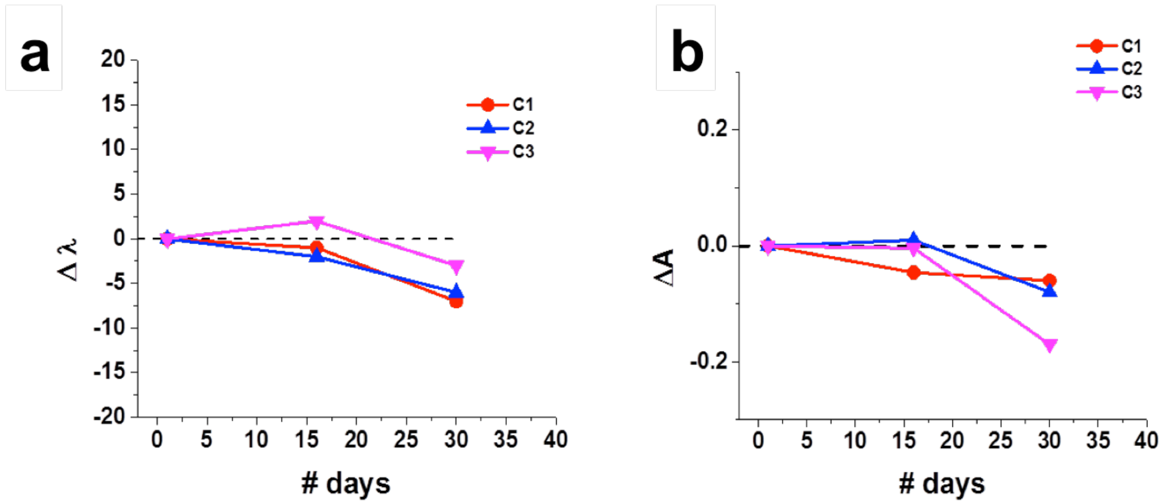

Supplementary Figure 5. Follow up UV-vis measurements of bulk HG/*x*NR PNIPAM-AuNR hydrogel samples. **(a)** Change of the absorption maxima from λ_{max}=813nm with time. **(b)** Change of the absorption intensity at λ_{max}=813nm with time. C1, C2, and C3 correspond to HG/10NP, HG/30NP and HG/50NR samples, respectively.

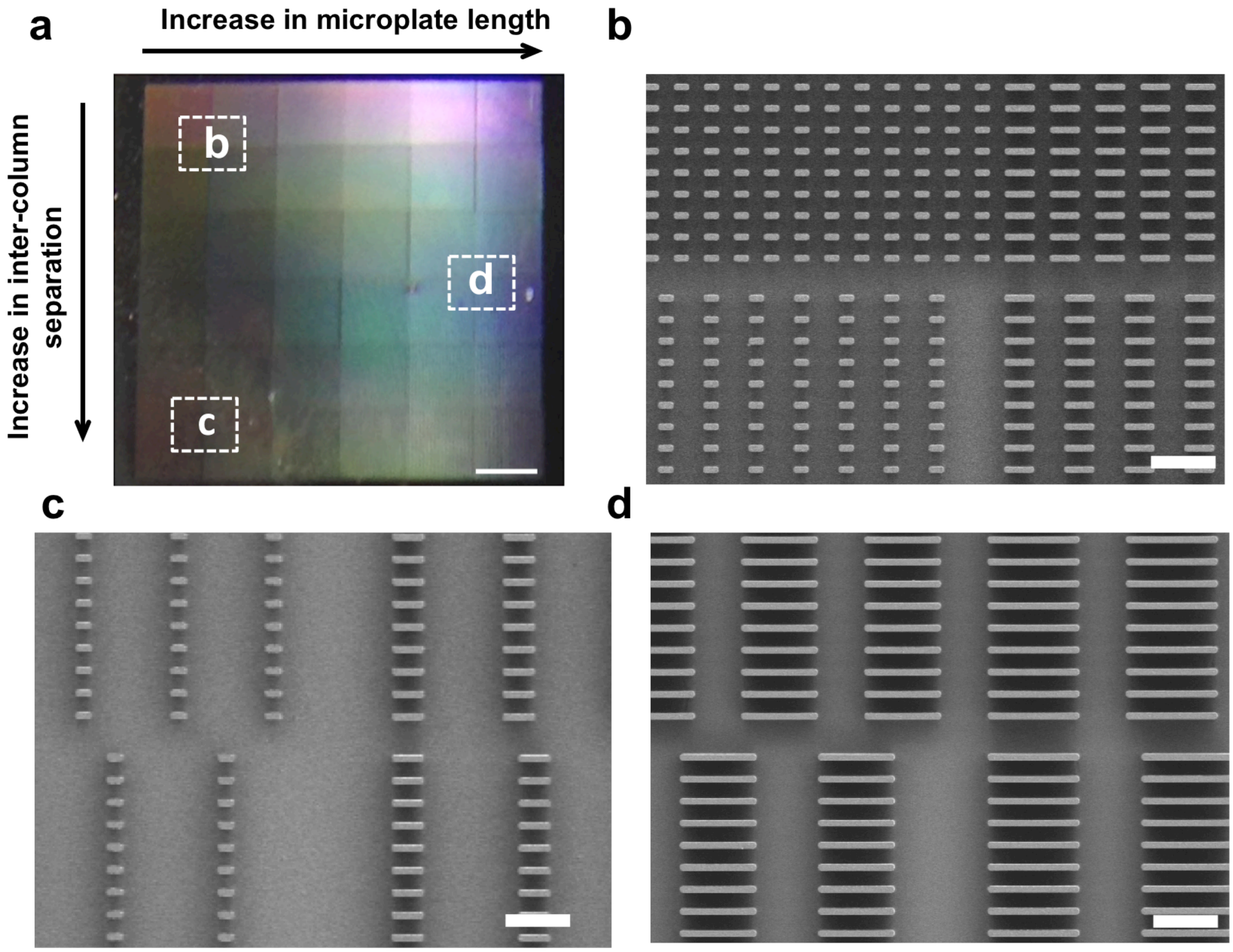

Supplementary Figure 6. Arrangement of the microstructures in the arrays (NOA61® replicas) used to fabricate the HAIRS-*x*NR. **(a)** Optical microscope image of a NOA61® microstructure array replica. The scale bar is 1mm. **(b-d)** SEM images of the microstructures taken at different locations in the microstructure array, which are marked with white dashed rectangles on (a). The scale bars are 10μm.

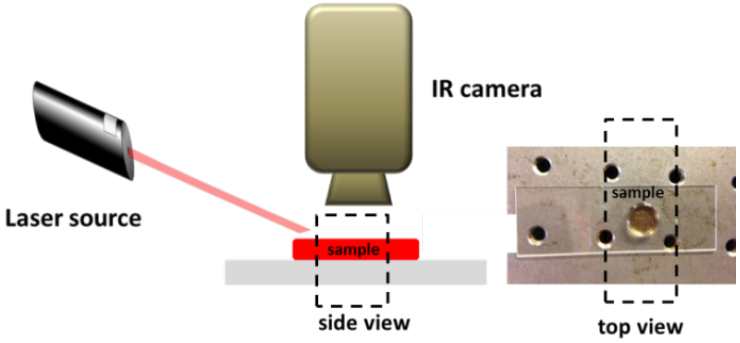

Supplementary Figure 7. Schematic representation of experimental setup for light triggered heating and photothermal phase transition of bulk composite PNIPAM-AuNR samples.

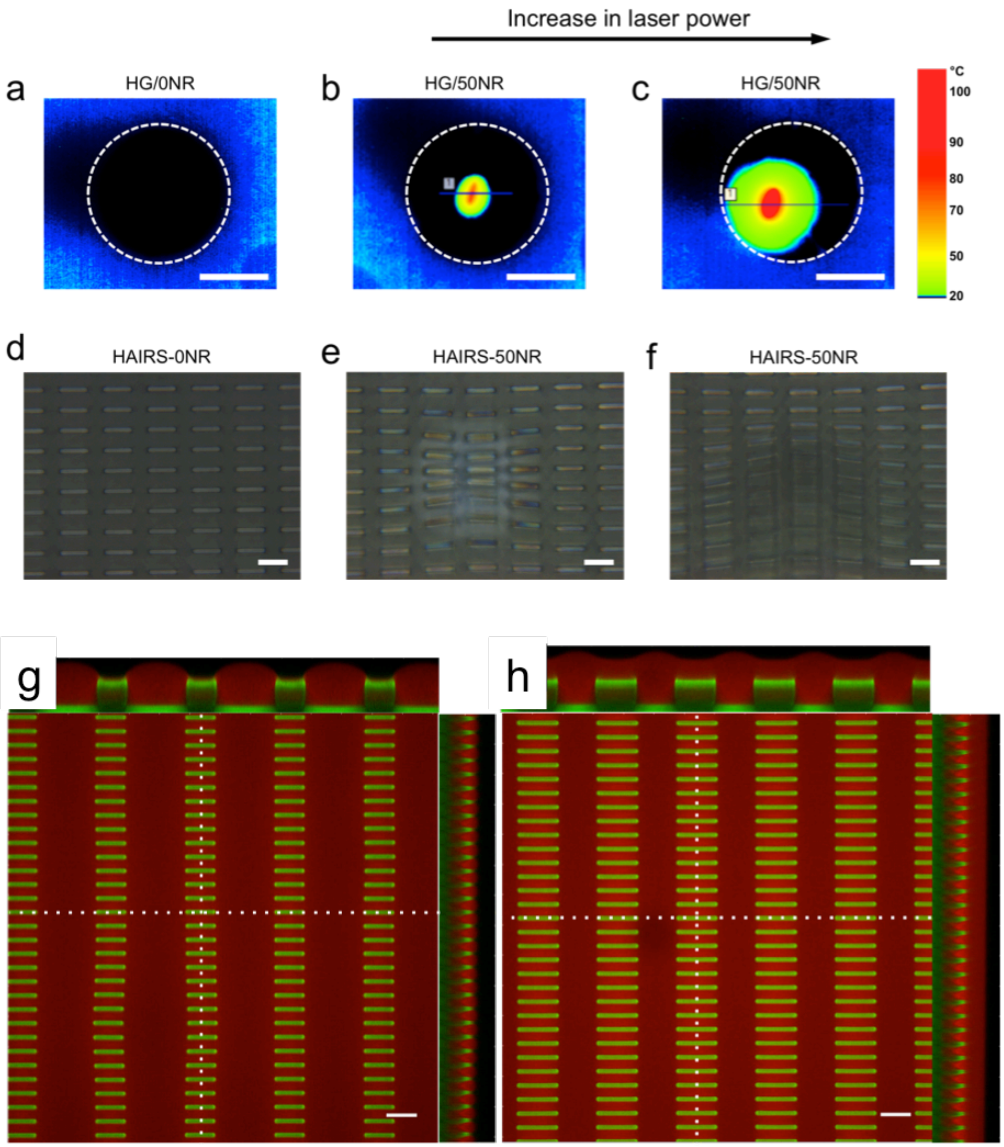

Supplementary Figure 8. (a-f) Effect of laser power on photothermal heating of the composite PNIPAAm-AuNR hydrogel in a **(a)** HG/0NR sample, **(b-c)** HG/50NR sample, **(d)** HAIRS-0NR sample (Movie 1), and a **(e-f)** HAIRS-50NR sample (Movie 2). The samples were irradiated at the following laser powers: (a-b) ~85mW; (c) ~280 mW; (d) ~11mW (e) ~4mW; (f) ~18mW. The exposure time was 5s. The dotted circles indicate the HG/NR sample boundaries. The local temperature increase is indicated by a rainbow color scheme in the infrared images. No photothermal response has been observed in (a) HG/0NR and (d) HAIRS-0NR samples irradiated with NIR laser light. **(g-h)** Reconstructed 3D confocal images from two different areas on a fluorescently labeled HAIRS sample showing submersion in some areas of the NOA61® microstructures by the PNIPAAm-AuNR hydrogel film. Green: microstructures (pyrromethene 546), red: pNIPAAm hydrogel (DY-485 XL). The white dotted lines mark the location of two cross-sections shown at the top and side of the top-view images. **(g)** The microstructure tips remain clear of hydrogel. **(h)** The microstructures in the right image are clearly submerged by a hydrogel layer that is several micrometers thick. The scale bars are (a-c) 2.5mm, (d-f) 10µm, and (g-h) 15µm.

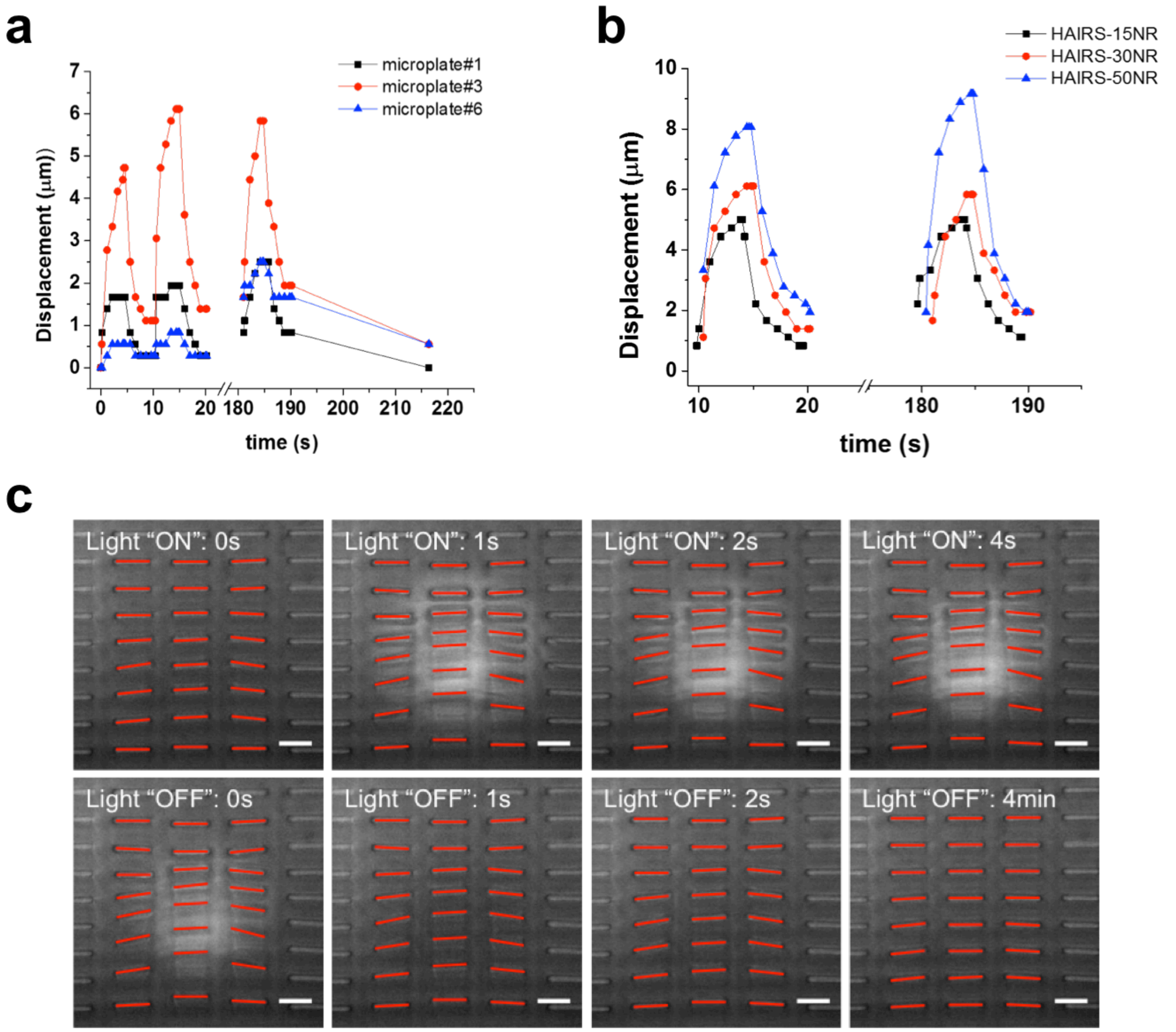

Supplementary Figure 9. (a) Microstructure tip displacement as a function of the distance from the central line where the bending direction of the microplates reverses. The movement of the microstructures #1, #3 and #6 (labeled in Figure 5b) over time in a HAIRS-30NR sample is shown (Movie 3). **(b)** Displacement as a function of the AuNR concentration. The movement of the microstructure #3 (labeled in Figure 5b) during a single actuation cycle for HAIRS-15NR, HAIRS-30NR, and HAIRS-50NR samples is shown. **(c)** Series of images taken from a movie sequence recorded during a cyclic actuation experiment on a HAIRS-30NR sample (Movie 5). The selected images were recorded at different time points before, during, and after the second actuation cycle in the series. The upper row shows the contraction of the gel and bending of the microstructures after the light (~4mW) is turned on. The refractive index of the hydrogel changes when it contracts. The bottom row of images shows the gel swelling and the microstructures returning to their upright positions after the light is turned off. The red bars indicate the positions of the microstructure tips. The scale bars are 10μm.

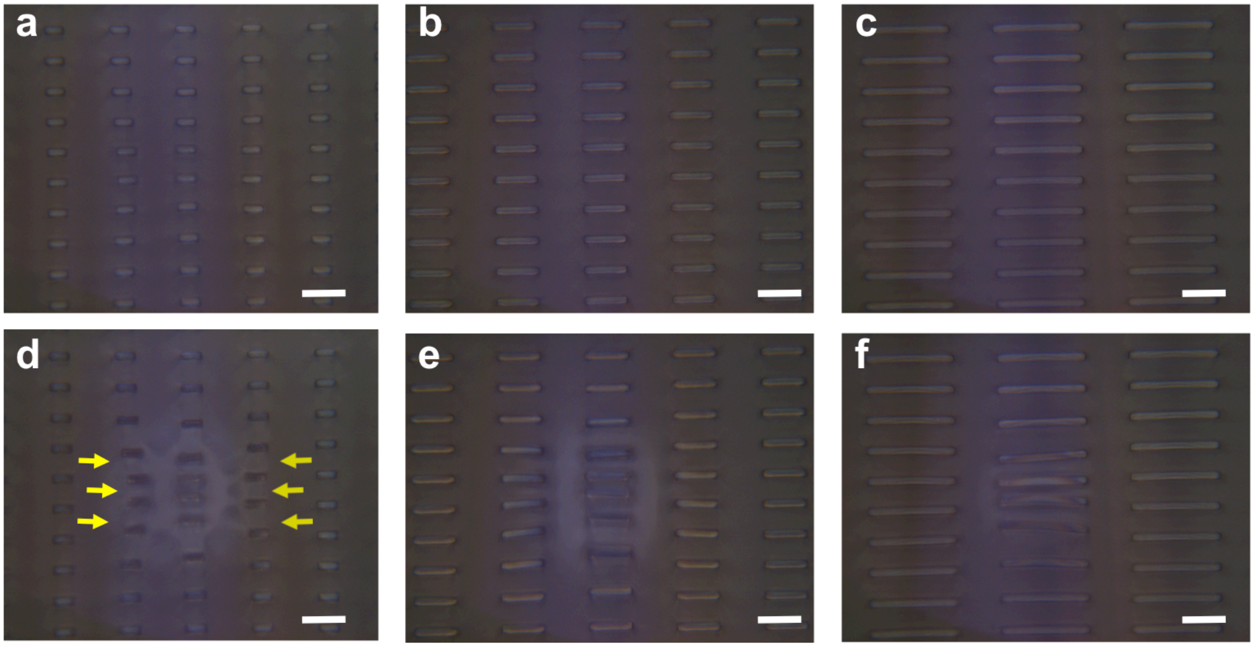

Supplementary Figure 10. Microstructure tip displacement as a function of the cross-sectional geometry. (a-c) Images of different locations on a HAIRS-30NR sample with (a) 2x5μm, (b) 2x10μm, and (c) 2x30μm rectangular microplate cross-sections, respectively. The columns of microplates are separated by 10μm, and the rows by 5μm. (d-f) Images of the same locations after the laser is turned on (~18mW). (d) The 2x5μm also bend along the axis of their longer dimension (Movie 6). (e) The 2x10μm and (f) the 2x30μm microplates bend uniaxially along the axis of their shorter dimension, with differential bending of different portions of the microplate being particularly evident as the microplate length increases (Movie 6). The scale bars are 10μm.

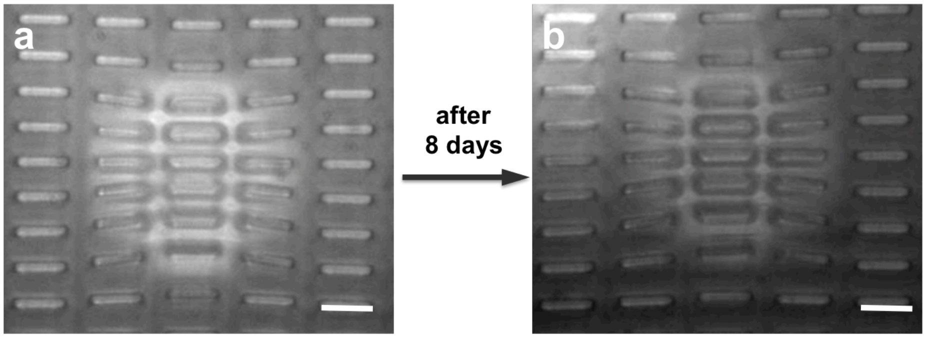

Supplementary Figure 11. Sample images taken from movie sequences recorded during two experiments set eight days apart, with repeated 5s "ON" (~18mW) and 5s "OFF" illumination cycles of the same location on a HAIRS-15NR sample (Movie 8). The scale bars are 10μm.

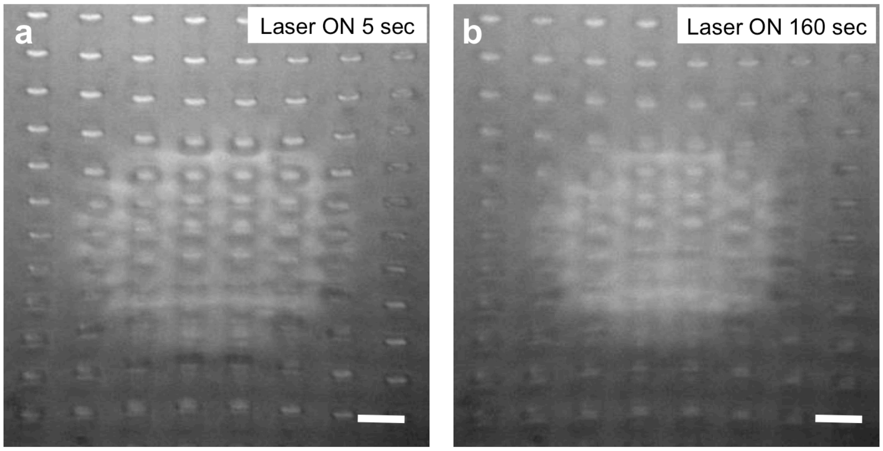

Supplementary Figure 12. Sample images taken from a movie sequence recorded during an experiment where a HAIRS-15NR sample was exposed to the laser (~49.5mW) continuously for ~2.5min (Movie 9). A shift in the focal plane from slight motions of the sample impacts the appearance of the sample and the spot size of the region where the hydrogel is contracted and has undergone a change in refractive index. Nonetheless, in both images the degree of displacement of the microstructures is constant. The scale bars are 10μm.

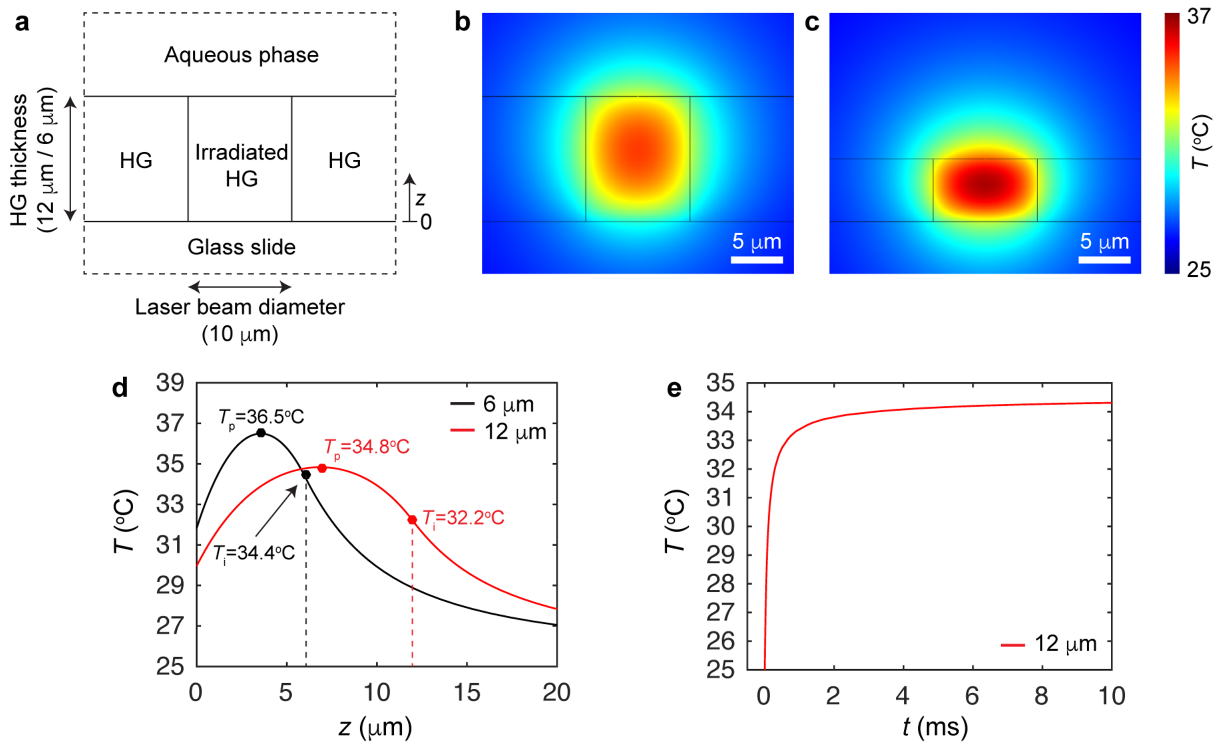

Supplementary Figure 13. (a) Scheme of the simplified sample geometry used in the simulations. **(b)** Simulated results for the temperature distribution after 5s of sample irradiation (non-collapsed hydrogel). **(c)** Simulated results for the temperature distribution after 5s of sample irradiation (collapsed hydrogel). **(d)** Simulated results for the temperature as a function of the distance from the bottom of the hydrogel layer for the two cases (non-collapsed and collapsed hydrogel). The peak temperatures (T_p) within the hydrogel layer and the temperatures at upper hydrogel interface (T_i) are indicated by dots and the corresponding values are displayed. **(e)** Simulated results for the temperature as a function of time inside the hydrogel film.

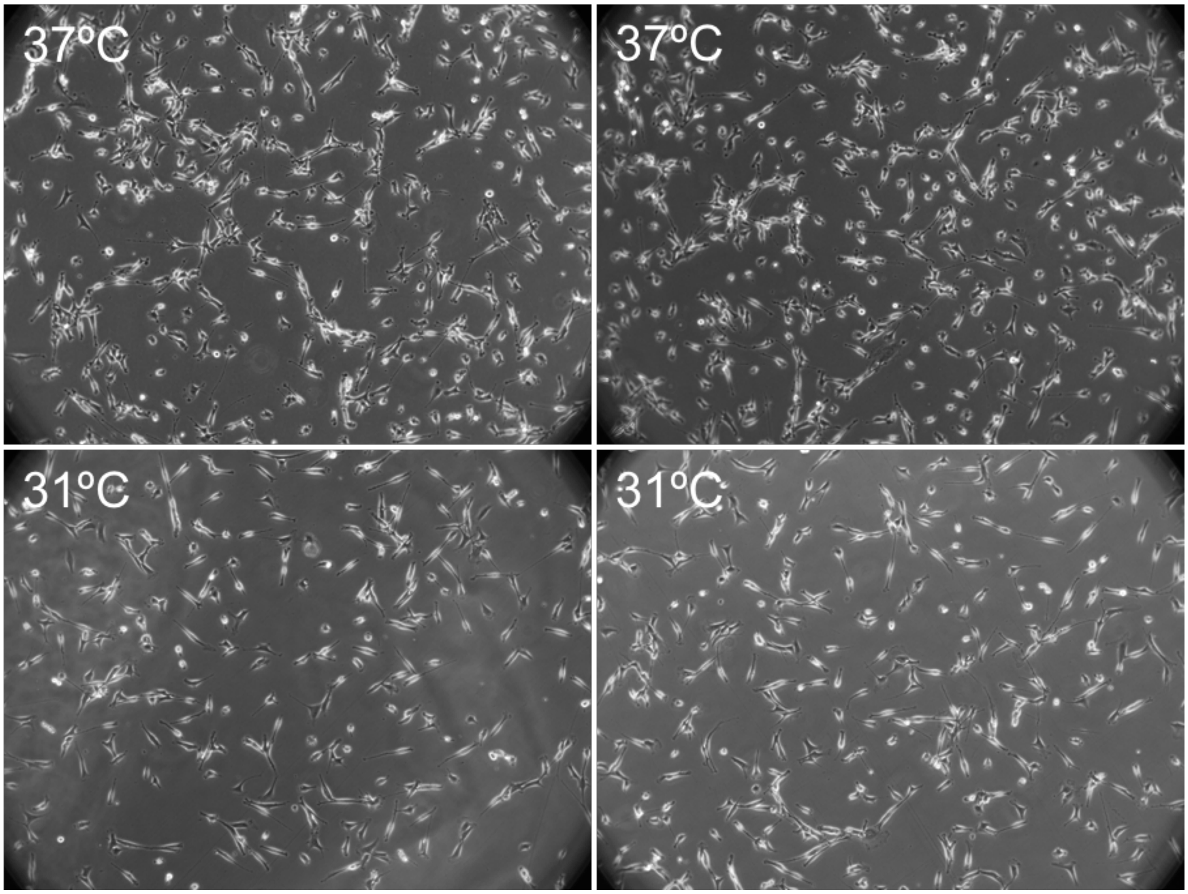

Supplementary Figure 14. Phase contrast images of the D1 ORL UVA cells seeded in regular tissue culture dishes (150k seeded per well in 6-well plates) and incubated at either 37°C (top images) or 31°C (bottom) for 24h before imaging (same incubation period as for the cells used in the mechanical stimulation experiments). The density of the cells is slightly lower in the dish that was incubated at 31°C, which indicates that the growth rate is lower at that temperature.

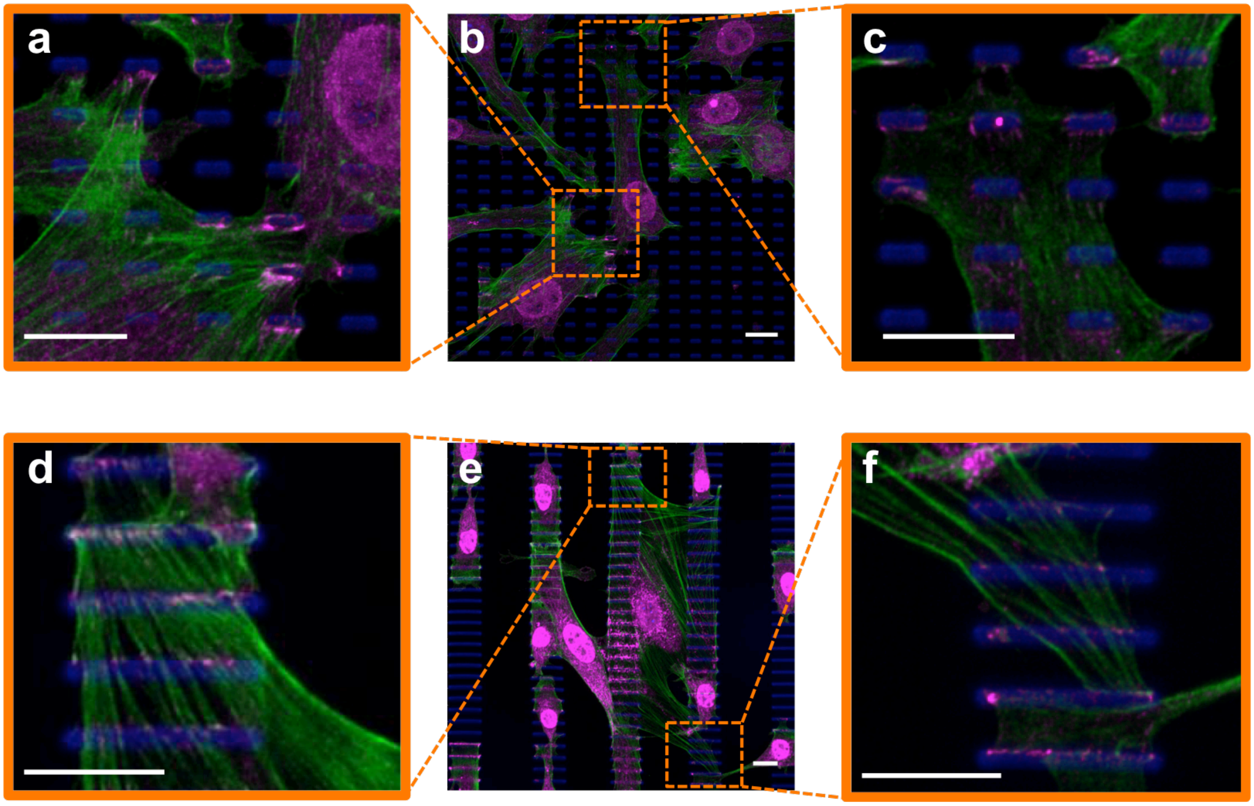

Supplementary Figure 15. Representative reconstructed 3D confocal images showing D1 ORL UVA cells that were grown on HAIRS samples at 31°C for 24h and were then fixed and immunostained for the focal adhesion protein vinculin. The close-up views clearly show that the cells form focal adhesions on the tips of the microstructures. Green: actin (phalloidin), magenta: anti-vinculin antibody (ab73412), blue: autofluorescence from the microstructures. The microstructure dimensions and spacing are: **(a-c)** 2μm-wide, 5μm-long, 5μm between neighboring rows, and 5 μm between neighboring columns, and **(d-f)** 2μm-wide, 20μm-long, 5μm between neighboring rows, and 30μm between neighboring columns. The scale bars are 15μm.

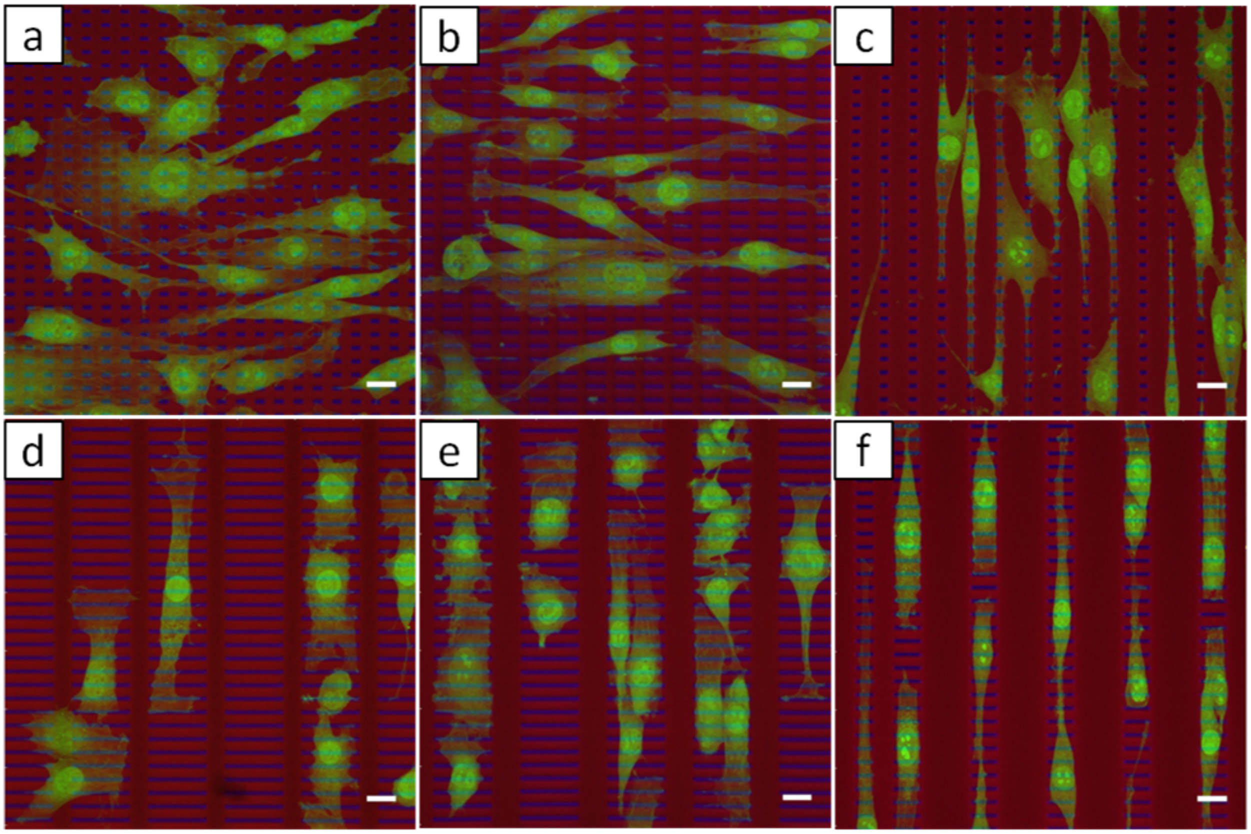

Supplementary Figure 16. Reconstructed 3D confocal images of cells attached to the tips of microstructures with different geometries and spacings in fluorescently labeled HAIRS samples, which show how cell alignment changes based on the geometric parameters of the underlying surface. The cells were grown at 30°C for 24h before fixing (green: D1 ORL UVA cells labeled with HCS CellMask Deep Red stain, blue: microstructures labeled with pyrromethene 546, red: PNIPAAm hydrogel labeled with DY-485 XL). The microstructure dimensions and spacing in the surface plane are: **(a)** 2μm-wide, 5μm-long, 5μm between rows, and 5μm between columns, **(b)** 2μm-wide, 10μm-long, 5μm between rows, and 5μm between columns, **(c)** 2μm-wide, 5μm-long, 5μm between rows, and 10μm between columns, **(d)** 2μm-wide, 30μm-long, 5μm between rows, and 10μm between columns, **(e)** 2μm-wide, 30μm-long, 5μm between rows, and 15μm between columns, **(f)** 2μm-wide, 10 and 15μm-long, 5μm between rows, and 10 and 25μm between columns. When the spacing between the columns of microplates increases from 5μm to 10μm, the cell alignment direction transitions abruptly from being parallel to the long axis of the microplates to being perpendicular to the long axis of the microplates. Notably, the cells are still able to bridge the distance at 10μm. The scale bars are 15μm.

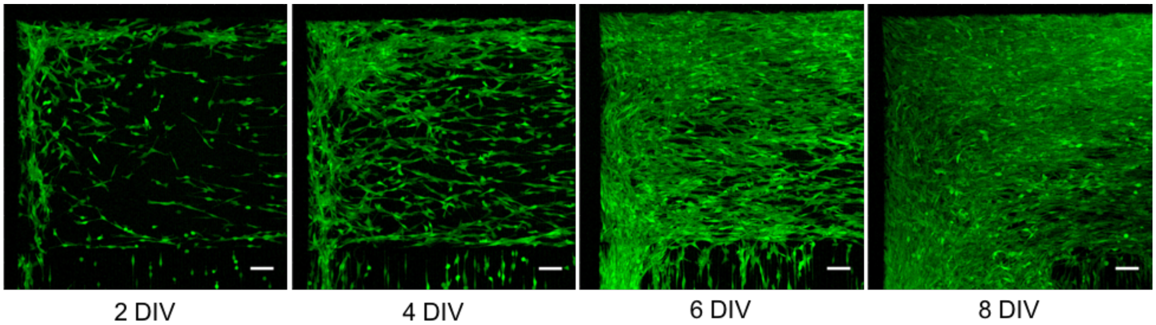

Supplementary Figure 17. Fluorescent images showing the proliferation of D1 ORL UVA cells over the course of a week on a HAIRS-50NR sample cultured at 31°C. The cells were genetically modified to express green fluorescent protein (GFP). The sample was imaged at (from left to right): 2 DIV (days *in vitro*), 4 DIV, 6 DIV, and 8 DIV. The area in the field of view is primarily covered by 2μm by 5μm microstructures, with 5μm separating the rows of microstructures, and 5μm separating the columns of microstructures, with a transition to 10μm separating the columns of microstructures at the bottom of the images. The cells reach confluency more rapidly when a larger fraction of the surface area is adhesive. The scale bars are 100μm.

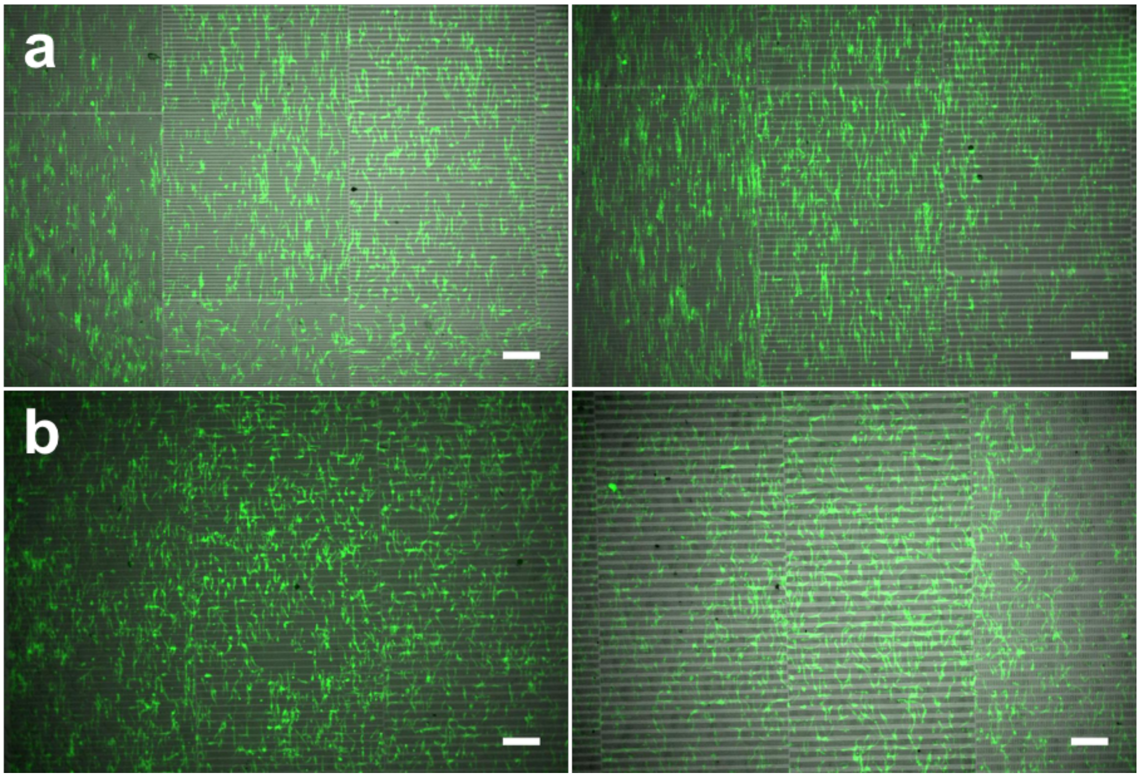

Supplementary Figure 18. Fluorescent images of D1 ORL UVA cells that were grown at 37°C for 24h on the ~12μm-high HAIRS microstructures without the PNIPAAm hydrogel. The cells (green) are labeled with CellTracker Green CMFDA, and a transmitted light image (grey) shows the columns of microstructures. **(a)** The microstructures are all 2μm-wide with 5μm between rows, while the other dimensions are varied (microplate length: 5-30μm, distance between columns of microplates: 5-15μm). **(b)** The microstructures are all 2x5μm microplates, arranged in columns of three closely spaced plates (5μm between rows and columns of the microplates) which are separated by distances varying from 10-30μm. The scale bars are 200μm.

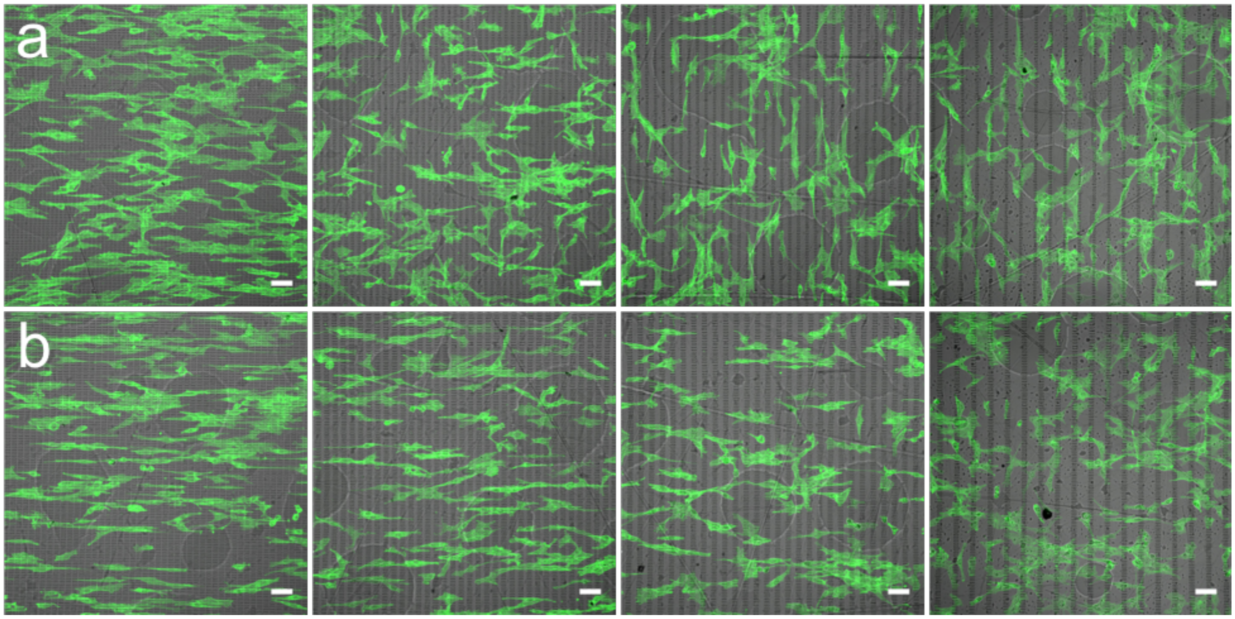

Supplementary Figure 19. Fluorescence images of fixed D1 ORL UVA cells that were grown at 37°C for 24h on 2µm-high microfeatures with the same dimensions and spacings in the surface plane as the HAIRS-*x*NR microstructures, as a control for creating a similar topography to the HAIRS-*x*NR surface plane, but with uniform adhesiveness over the surface (absence of the PNIPAAm). The cellular actin (green) is labeled with phalloidin, and a transmitted light image (grey) shows the columns of microfeatures. The microfeatures are all 2µm-wide with 5µm between rows, while the other dimensions are varied. **(a)** 5µm-long microfeatures with from left to right: 5µm, 10µm, 15µm, and 30µm between columns. **(b)** 10µm-long microfeatures with from left to right: 5µm, 10µm, 15µm, and 30µm between columns. The scale bars are 50µm.

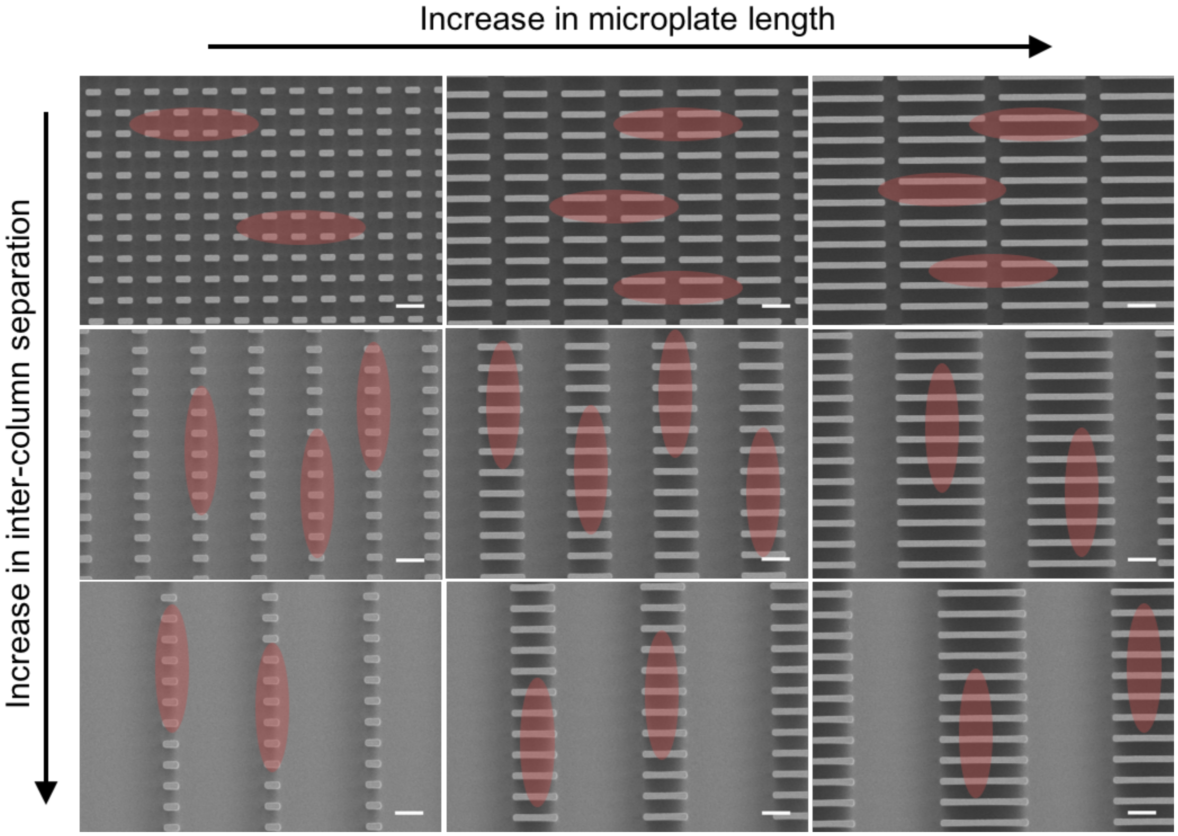

Supplementary Figure 20. Selected scanning electron microscopy (SEM) images showing a sampling of the different microstructure topographies tested on a single HAIRS/*x*NR sample, and the expected cell alignment directions. A single HAIRS microstructure array contained 36 distinct topography conditions, arranged in a continuous grid of microplates with each condition remaining constant over a $1mm^2$ area. The microplates were all 12μm-high and 2μm-wide. Their length was varied incrementally by 5μm between neighboring conditions going from left to right in the sample (5-30μm). The distance separating columns of microplates was varied incrementally by 5μm between neighboring conditions going from top to bottom in the sample (5-30μm). The distance separating rows of microplates was kept constant at 5μm over the entire sample. At some transition point in the spacing between columns of microstructures, the cells were expected to change their alignment direction and align with the short microplate axis, as is illustrated with the red ovals. The scale bars are 10μm.

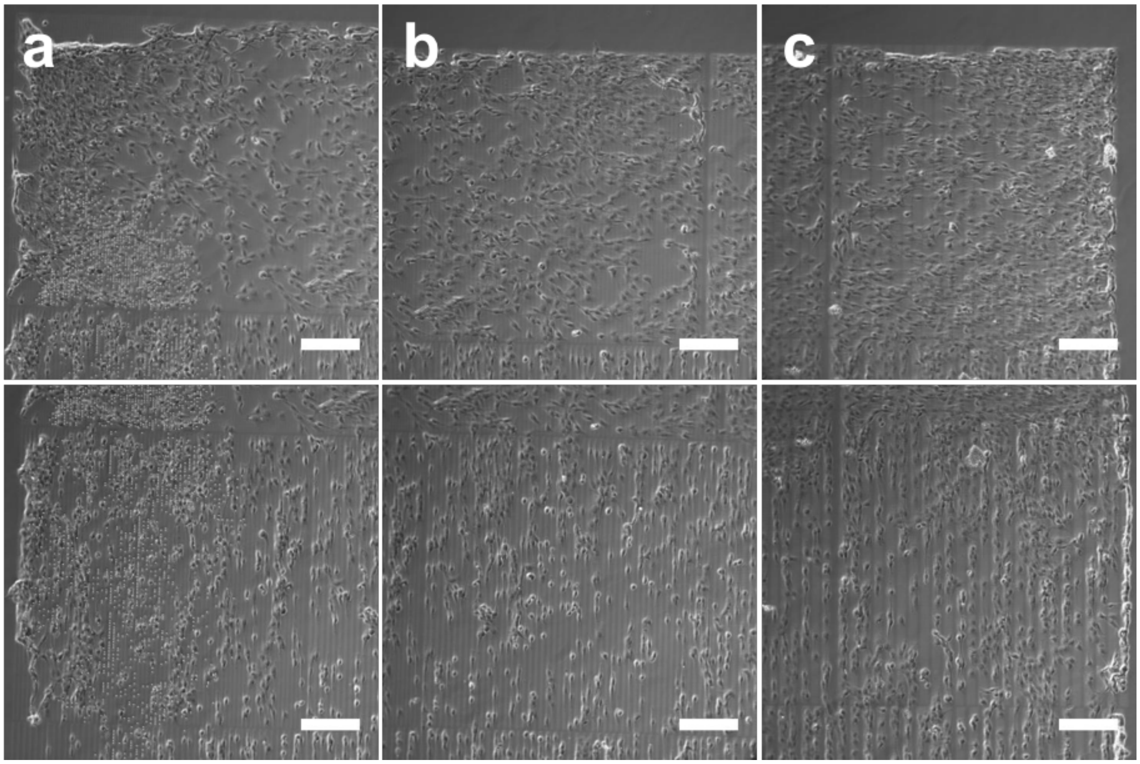

Supplementary Figure 21. Phase contrast images of fixed D1 ORL UVA cells that were grown at 37°C for 48h on a flat patterned gold film functionalized with a non-adhesive mPEG-thiol (MW 5000), where the adhesive areas have the same dimensions and spacings in the surface plane as the HAIRS-*x*NR microstructures, as a control for creating a similar adhesive pattern to the HAIRS-*x*NR surface plane, but with a flat surface and without the PNIPAAm hydrogel. The patterns of cell alignment observed on the HAIRS-*x*NR samples incubated at 31°C are reproduced on these samples with the cells incubated at 37°C.The columns of adhesive micro-islands are clearly visible in the areas of low cell density. The adhesive islands are all 2μm-wide with 5μm between rows, while the other dimensions are varied: **(a)** 5μm-long micro-islands with 5μm (top) or 10μm (bottom) between columns, **(b)** 10μm-long micro-islands with 5μm (top) or 10μm (bottom) between columns, **(c)** 30μm-long micro-islands with 5μm (top) or 10μm (bottom) between columns. The scale bars are 200μm.

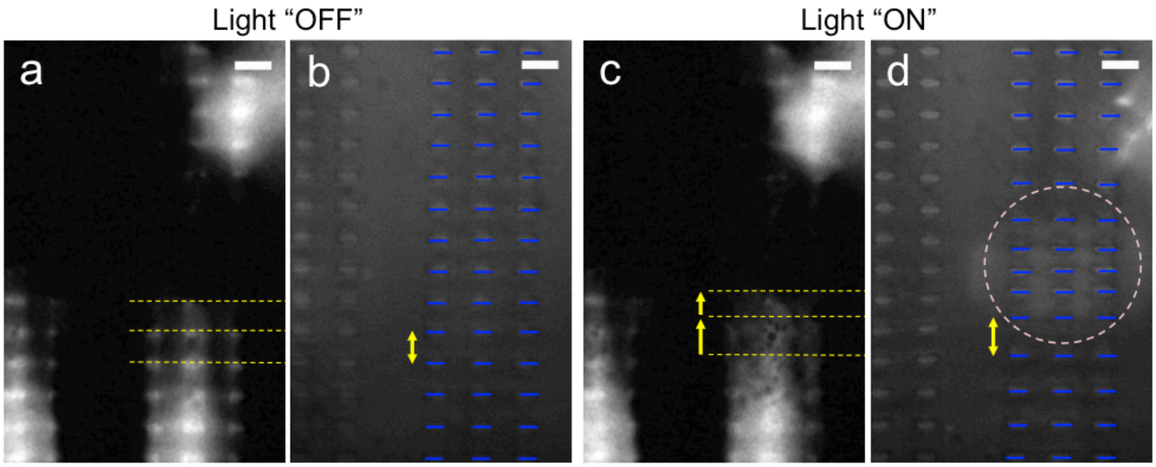

Supplementary Figure 22. Single cell micro-manipulation with targeted actuation of the microplates. Images taken from a movie sequence (Movie 11) recorded during a cell micro-manipulation experiment showing epifluorescence images of the cells on the left and reflected brightfield images of the underlying microstructures on the right. The cells were cultured on the HAIRS-15NR sample for 24h prior to the experiment. The laser (~4mW) is focused at the center of the pink dashed circle that outlines the area where the hydrogel is contracted and the microplates are actuated. The cells are labeled with CellTracker Green CMFDA. Images of two cells before the start of the experiment **(a)** and of the same cells approximately 3s after the laser is turned on **(c)** are shown. The cell on the bottom undergoes a significant change in shape due to the movement of the microstructures underneath it (**b** and **d**). The locations of two rows of microstructures at both time points are marked by the yellow dashed lines, and the elongation from 9μm (**b**) to 10.5μm (**d**) of the portion of the cell overlying that region is indicated by the yellow arrows. The blue bars in brightfield images mark the locations of the microstructure tips, and highlight the change in the distance separating the tips of adjacent microstructures after the hydrogel contraction is triggered. All scale bars are 10μm.

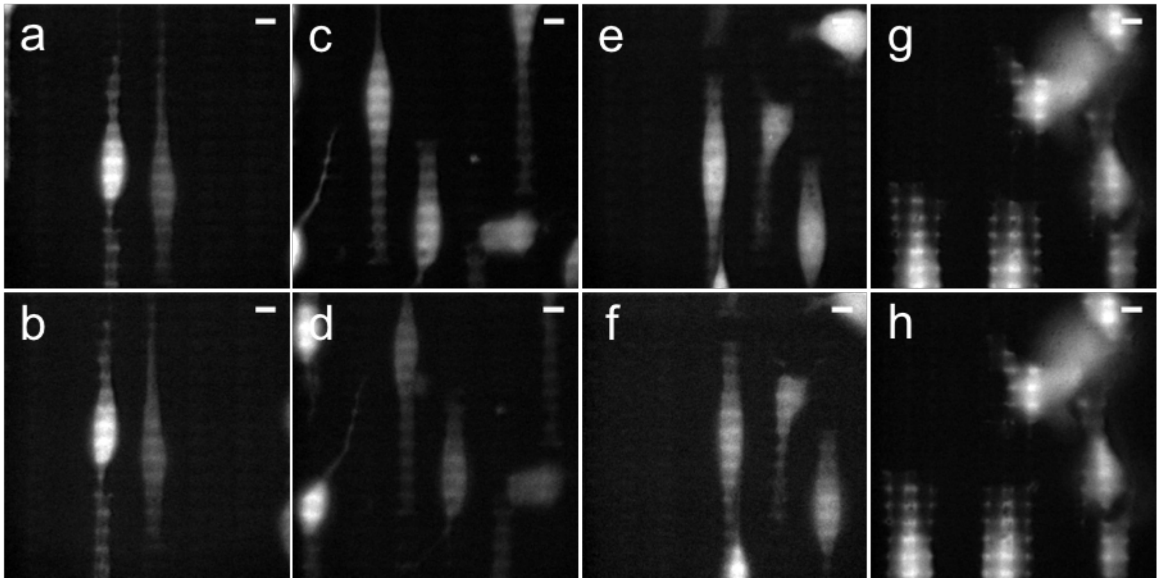

Supplementary Figure 23. Images taken from several movie sequences recorded during multiple cell micro-manipulation experiments showing epifluorescence images of the cells immediately before (top row) the mechanical manipulation experiment, and corresponding images taken immediately after the end of the experiment (bottom row). The cells were cultured on the HAIRS-*x*NR samples for 24h prior to the experiment, and were stained with CellTracker Green CMFDA. The mechanical strain profiles were: **(a-b)** 5 pulses of 3s each (2x ~18mW, 1x ~11mW, and 2x ~4mW, Movie 10), **(c-d)** fifteen short pulses and 5min of continuous strain (~4mW, Movie 12), **(e-f)** 6min of 3s pulses followed by 3s relaxation periods (~4mW, Movie 13), and **(g-h)** 5min of continuous strain (~4mW, Movie 11). All scale bars are 10μm.

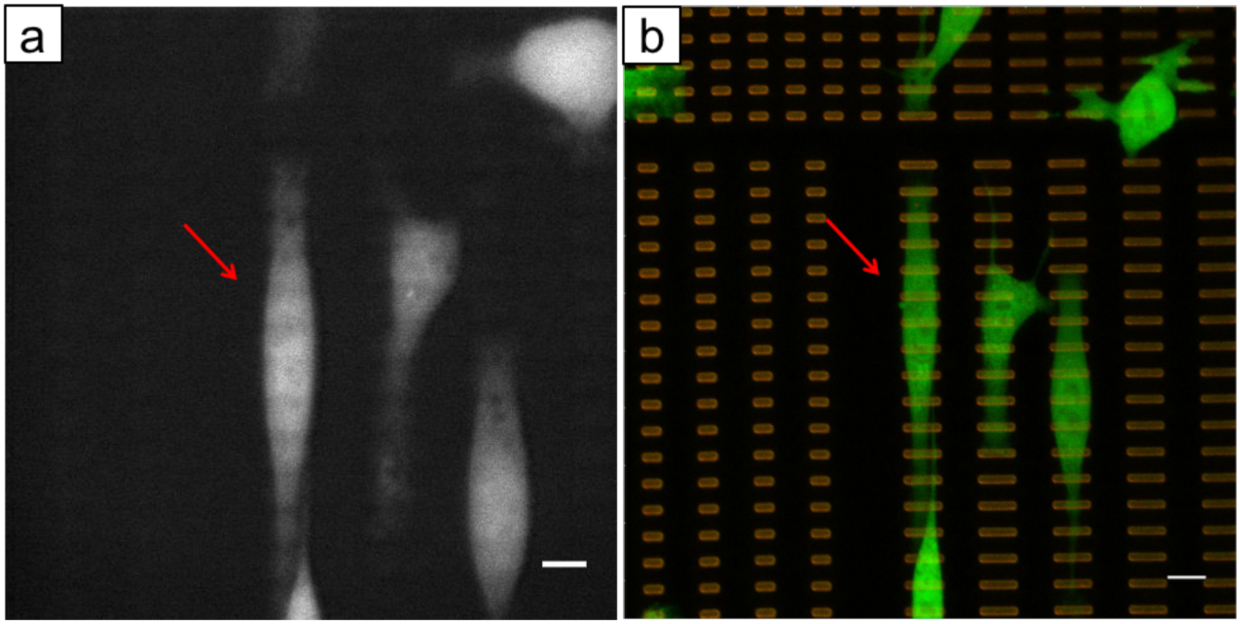

Supplementary Figure 24. Sytox Orange assay for cell viability after a mechanical manipulation experiment. **(a)** Epifluorescence image of a group of cells taken before the central cell (pointed at by the red arrow) was subjected to any mechanical strain or exposure to the actuation light. The cells are stained with CellTracker Green CMFDA. **(b)** Reconstructed 3D confocal image of the same cells after a Sytox Orange Nucleic Acid Stain cell viability assay that was performed 2h after the mechanical stimulation experiment (6min of 3s pulses followed by 3s relaxation periods at ~4mW). The Sytox Orange Nucleic Acid Stain (orange) did not noticeably penetrate the membranes of any of the cells (green), although it did stick to the microstructures. The scale bars are 10μm.

Supplementary Tables

Supplementary Table 1. Swelling ratios of HG/AuNR bulk samples with different concentrations of AuNRs. Measurements were performed for 3 different batches of samples to ensure reproducibility.

	W_s (mg) at 21°C	W_c (mg) at 45°C	*SR*
**HG/0NR**	172.0±6.1	70.7±1.5	1.43±0.03
**HG/10NR**	178.3±2.1	72.7±2.5	1.46±0.06
**HG/20NR**	179.0±2.6	78.3±6.0	1.30±0.21
**HG/30NR**	178.1±2.6	74.3±3.5	1.40±0.08
**HG/40NR**	176.2±2.5	72.7±5.1	1.43±0.17
**HG/50NR**	181.3±2.4	76.7±8.1	1.38±0.24

Supplementary Table 2. Material parameters used in the heat generation and transfer simulations

**Material parameters**	ρ (kg m^{-3})	c_P (J kg^{-1} K^{-1})	k (W m^{-1} K^{-1})
HG / aqueous phase	1000	4181	0.58
Glass	2203	703	1.38

Supplementary Notes

Supplementary Note 1. Determination of the AuNR concentration

The accurate calculation or estimation of the molar concentration of AuNRs is not trivial.[1,2] The synthesis of AuNRs is less facile than that of gold nanoparticles (AuNPs). Unlike the synthesis of spherical nanoparticles, the growth of AuNRs requires weak reducing agents, leading to an unknown degree of gold reduction. Moreover, the variability in AuNR dimensions, low yield of the desired shape/size, and side products of unwanted nanoparticle shapes could contribute to an inaccurate determination of the AuNR concentration. Our approach is based on two steps. First, we tried to obtain very monodisperse samples of AuNRs by repeated washing and centrifugation to reduce the polydispersity in the size and shape of the AuNRs. Different fractions of AuNRs were analyzed by TEM and UV-vis measurements to find the appropriate conditions to obtain samples with optimum monodispersity. The optimized sample, based on TEM and UV-vis analysis, was analyzed by Inductively Coupled Plasma – Atomic Emission Spectroscopy (ICP-AES) in order to determine the amount of gold per unit volume. This, in conjunction with the UV-vis absorption of the AuNR solution and the average volume of the AuNRs found from the TEM measurements (Supplementary Fig.2b), was used to determine the extinction coefficient ε. This extinction coefficient was used in the calculation of the AuNR concentration in other batches and in the hydrogel samples. The AuNRs were considered to have the geometry of a cylinder capped with two hemispheres (Supplementary Fig.2b). The average volume of the AuNRs (V_{NR}) can thus be calculated using the following formula:

$$V_{NR} = \frac{\pi D^2}{12}(3L - D)$$

Supplementary Equation 1.

where L is the length and D is the diameter of an AuNR measured from TEM images using ImageJ software. The statistical analysis of ~150 AuNRs gave the following numerical outcomes for L and D: L = 37.1±2.2nm, D = 9.1±0.8nm. This results in an aspect ratio R of ~4. By substituting these values into Supplementary Equation 1, we get: $V_{AuNR} = 2.2 \times 10^{-18}\mathrm{cm}^3$.

From ICP measurements we determined W_{Au}, the concentration of Au ions in the solution, to be 62.2μg ml^{-1}. The total volume of gold $V_{AuTotal}$ in 1ml is thus:

$$V_{AuTotal} = W_{Au}/\rho_{Au} = 62.2\times10^{-6}g/19.3g\ cm^{-3} = 3.22\times10^{-6}cm^{-3}$$

Supplementary Equation 2.

where ρ is the density of gold. The number of AuNRs is therefore:

$$N_{AuNRs} = V_{AuTotal}/V_{AuNR} \approx 1.46\times10^{12}$$

Supplementary Equation 3.

The number of moles of AuNRs in 1ml is:

$$mol_{AuNR} = N_{AuNRs}/N_A = 1.46\times10^{12}/6.02\times10^{23} = 0.24\times10^{-11}mol$$

Supplementary Equation 4.

where N_A is Avogadro's number. Thus, the estimated concentration of AuNRs in the solution is:

$$C_{AuNR} = mol_{AuNR}/0.001L \approx 2.4nM$$

Supplementary Equation 5.

According to the Beer–Lambert law:

$$\varepsilon = \frac{A}{lC}$$

Supplementary Equation 6.

where A is the absorbance (optical density) of the solution, ε is the extinction coefficient (M^{-1} cm^{-1}), l is the length of the light path (cm) through the solution, and C is the concentration (M). From the UV-vis data analysis of the AuNR stock solution, we estimated that $\varepsilon = 1.15\times10^{10} M^{-1} cm^{-1}$ at the longitudinal plasmon resonance wavelength (815nm). The concentration of AuNRs in the hydrogel samples was estimated from their absorption at 815nm using the extinction coefficient calculated above and with the length of the light path equal to 0.12mm.

Supplementary Note 2. Homogeneous heating effect on the optical properties of bulk AuNR-HG samples

The composite HG/*x*NR bulk samples studied in this work display a reversible shift in the surface plasmon band of the AuNRs during heating/cooling cycles (Supplementary Fig.3). Before recording the high temperature spectra, the bulk HG/*x*NR samples were placed in an oven set to 45°C for ~12h. An obvious change of volume was observed when the temperature was varied between 25°C and 45°C (Supplementary Fig.3a). As the temperature increases above the LCST of PNIPAM hydrogels (~32°C), the collapse of the hydrogel produces broadening and causes a red shift of ~10nm of the surface plasmon band (Supplementary Fig.3b). The red shift is due to the near-field coupling between adjacent AuNRs when they are brought close together, and also the refractive index change of the hydrogels at transition temperature.

Supplementary Note 3. Morphological characterization of PNIPAAm-AuNR bulk samples

A set of samples with different AuNR concentrations (HG/30NR and HG/50NR) were cured in Eppendorf cups and swelled in ethanol for at least 24h. Then the samples were cut into strips, transferred from ethanol to a 50/50 mixture of ethanol/acrylic resin (LR White, London Resin Co.) at room temperature. The samples incubated at RT until they sank to the bottom of the vial

(~12h). The samples were then transferred into neat resin solution and allowed to incubate for 24h. Pieces were transferred into gelatin capsules using a transfer pipet with the tip cut off to enlarge the opening. Paper labels were added, the capsules were topped off, then tightly sealed. The resin was polymerized at 50°C overnight. Samples were cut on a Leica Ultracut UCT Ultramicrotome. Sections were collected with TEM grids at several thicknesses ranging from 90nm to 300nm, dried and kept for TEM analysis. Supplementary Figure 4 shows representative TEM images of PNIPAAm-NR bulk samples (HG/50NR).

Supplementary Note 4. Stability of PNIPAM-AuNR bulk samples (aging effect)

On the basis of previous reports regarding the relationship between the network pore size and the diffusion of nanoparticles in gels, it is anticipated that the AuNRs (diameter ~10nm, length ~37nm) will probably experience difficulty in diffusing through the PNIPAAm gel network (BIS content: 1mol%, expected pore size <10nm) due to their asymmetric shape.[3] Moreover, the modification of AuNRs with PEG adds additional thickness to the NR dimensions (for MW_{PEG} = 5000 the film thickness is ~6nm based on the random coil orientation), which further decreases the already poor mobility of the AuNRs within the gel. Indeed, the absorption spectrum of the bulk hydrogel samples containing AuNRs measured after ~1 month of storage showed almost no shift in band position, and less than 10% loss of 813nm absorption intensity. These results indicate stable incorporation of the AuNRs and almost no diffusion out of the gel (Supplementary Fig.5).

Supplementary Note 5. Swelling ratios of PNIPAAm and PNIPAAm-AuNR bulk samples

The effect of AuNR incorporation on the swelling behavior of PNIPAAm hydrogel has been examined. The bulk PNIPAAm samples prepared as described above with various concentrations of AuNRs were kept in DIW at room temperature for at least 48h to ensure full equilibration of the swelling process. After such equilibration, the fully swollen gels were taken out of the water, tapped with filter paper to remove excess water from the sample surface and weighed. Subsequently, samples were returned to water and introduced into an oven set at 45°C for 48h. After equilibration at this temperature, shrunken gels were taken out of the water, wiped with tissue paper and weighed again. Although the time taken for measurements was minimized and gels were wiped to remove any adherent water, changes in temperature during the process of measurement and the presence of water residues can be sources of error in the swelling ratio. The swelling ratio of samples, *SR*, was calculated according the following formula:

$$SR = \frac{W_s - W_c}{W_c}$$

Supplementary Equation 7.

where W_s and W_c were the weight of the swollen and contracted samples, respectively.[4] Measurements were performed for 3 different batches of samples to ensure reproducibility. The data is summarized in Supplementary Table 1. The shrinkage in the volume of all examined

hydrogels was observed when the temperature was changed from 21°C to 45°C. The swelling ratios were found to be similar between PNIPAAm hydrogel and composite PNIPAAm-AuNRs samples. This indicates little or no influence of AuNRs incorporation on the swelling behavior of PNIPAAm hydrogels within experimental concentrations range. These results are somewhat supported by morphological TEM analysis of bulk samples showing not dense, sparse distribution of AuNRs in gels even at highest concentration used in our experiments (HG/50NR).

Supplementary Note 6. Light-triggered heating and photothermal phase transition of bulk and thin film PNIPAAm-AuNR samples

The bulk hydrogel samples were slightly dried with filter paper and placed on the glass microscope slide. The laser diode was mounted at ~45° from the sample plane at distance of ~10cm. The laser beam (d ~1mm) was focused at the center of each sample (Supplementary Figs.7,8a-c). The HAIRS-*x*NR samples were simultaneously imaged and exposed to the NIR laser light in the set-up described in the main text (Supplementary Fig.8d-f). The thickness of the PNIPAAm-AuNR hydrogel film in the HAIRS-*x*NR samples made by the described procedure (Fig.2b, Methods) was examined by fluorescently labeling both the microstructure prepolymer and the hydrogel in some samples, and then imaging with a confocal microscope (Supplementary Fig.8g-h). The hydrogel film was seen to submerge the microstructure tips on some areas of the sample surface, but not others. The local thickness of the hydrogel film has an impact on the actuation of the microstructures, and also on cell adhesion (the cells do not adhere to PNIPAAm in its swollen phase).

Supplementary Note 7. Microstructure movement during an actuation cycle

The data used for the graphs in Figure 5c-d of the manuscript came from movie sequences collected during light-induced actuation experiments (Movies 3 and 4 for HAIRS-30NR and HAIRS-50NR respectively). In each data point image, the displacement of the center of the selected microstructure was measured with respect to its starting position. During the experiment, the actuation light was cyclically turned on for ~5s and then turned off for ~5s. The microstructures would stabilize in a relaxed state several seconds after the light was turned off, but would eventually return to a fully upright position several minutes after the last actuation cycle (Supplementary Fig.9).

The motion of the microplate tips during an actuation cycle strongly depends on the geometry of their cross-sections. In our experiments, the region where the hydrogel contracts is roughly circular in the 2D plane of the HAIRS surface, and therefore the forces generated by the contracting polymer network involve a strong radial component. The result is that microplates with lower aspect ratio cross-sections will bend along both dimensions towards the center of the contracted area (Supplementary Fig.10a,d). On the other hand, longer microplates resist deformation along their longer dimension, and therefore all bend along a single axis (Supplementary Fig.10b,c). When very long microplates are used, it becomes possible to displace the center of an individual microplate without displacing the two ends (Supplementary Fig.10c,f).

Supplementary Note 8. Estimation of the force required to deflect a single microstructure

The contraction of the PNIPAAm-AuNR hydrogel generates sufficient force to cause the bending actuation of the embedded microstructures (Figs.4,5). The amount of force required to bend a single microstructure, which can be used as an estimation of the force transmitted to the cell via the focal adhesions on a single microstructure, can be estimated by:

$$F = D\frac{3E}{h^3}I, \text{where } I = \frac{lw^3}{12}$$

Supplementary Equation 8.

where D is the distance by which the tip of the microplate is deflected (μm), E is Young's (elastic) modulus of the microstructure polymer material (GPa); h is the height of the microplate (μm); I is the area moment of inertia (geometry-dependent) for a beam with a rectangular cross-section (m^4); w is the width of the microplate (μm), and l is the length of the microplate (μm).[5,6] For a microplate made out of NOA61® with dimensions of h=12μm, w=2μm and l=10μm, the magnitude of force required to bend and displace the tip by 8μm can be estimated to be ~100μN (Fig.5c).

Supplementary Note 9. Stability over time of the photothermally-triggered actuation response of the HAIRS-*x*NR material

The ageing of the HAIRS-*x*NR material was examined by testing the response of the microstructures at a single location on the sample surface to cyclic actuation at two time points spaced eight days apart. In the interim, the samples were stored in water at room temperature. We did not observe any noticeable differences between the material's performance at the two time points (Supplementary Fig.11, Movie 8).

Supplementary Note 10. Heat generation and diffusion in the HAIRS-*x*NR

Successful manipulation of live mammalian cells with our method relies on two criteria: 1) the temperature of the PNIPAAm hydrogel (HG) is raised above the hydrogel transition temperature and 2) the temperature of the cells is kept below the upper physiological limit (~39°C). Thus, three-dimensional (and time-dependent) information of the temperature within the sample, and especially at the cell interface is required. Direct measurement of the temperature in such a small volume is difficult, although our results show that shortly after being exposed to the NIR laser, the system reaches a steady state where the size of the actuated area stops increasing, indicating that the temperature distribution is no longer changing (Supplementary Fig.12, Movie 9). Hence, we solved the heat equation numerically with the finite element method (COMSOL 5.2).

For simplicity, we neglected the microstructures and simulated heat generation and diffusion in and out of a semi-infinite HG film (height h=12μm) sandwiched between a thick glass slide and water (Supplementary Fig.13a). The heat equation given throughout the simulated volume is given by:

$$\rho c_P \frac{\delta T}{\delta t} - \nabla \cdot (k \nabla T) = Q$$

Supplementary Equation 9.

where ρ is the density, c_P is the specific heat capacity, T is the temperature, t is the time, k is the thermal conductivity and Q is the volumetric heat flux. The material parameters used in the simulation are given in Supplementary Table 2. The volumetric heat flux Q is zero outside the volume heated by the NIR laser. Inside the heated volume Q depends both on the AuNR concentration and the laser power, and is calculated below. The concentration of AuNRs in the HG film was c=0.48nM (corresponding to the HG50 concentration used in experiments). The incident laser power was P_0=22.5mW, and the beam was assumed to uniformly irradiate a cylindrical volume in the HG film with a diameter of $2r$=10μm. According to the Beer-Lambert law, the absorbed optical power is given by:

$$P_{abs} = P_0(1 - 10^{-\varepsilon ch})$$

Supplementary Equation 10.

where $\varepsilon = 1.15 \times 10^{10} M^{-1}\ cm^{-1}$ is the extinction coefficient of the AuNR (see Supplementary Note 1). In the simulated conditions, $P_{abs} \approx 0.34$mW, indicating that only ca. 1.5% of the incident laser power is converted into thermal energy. We further assume that the absorbed power is uniformly distributed over the cylindrical volume, yielding a volumetric heat flux of :

$$Q = \frac{P_{abs}}{V} = \frac{P_{abs}}{\pi r^2 h} \approx 3.6 \cdot 10^{11}\ W\ m^{-3}$$

Supplementary Equation 11.

Using these parameters, the results for the temperature distribution in the sample after 5s of irradiation is shown in Supplementary Figure 13b. The temperature is highest approximately in the mid-plane of the HG film, and decreases both towards the upper interface of the HG film (HG-water interface in close proximity to the basal plane of the cells) as well as towards the solid support. The highest temperature reached is 34.8°C, and the temperature at the HG-water interface is 32.2°C (Supplementary Fig.13d). If we assume that the HG collapses by 50% due to this heating (effectively reducing the HG film thickness to 6μm and doubling the AuNR concentration), the temperature inside the HG film rises slightly (Supplementary Fig.13c). In the collapsed state, the highest temperature inside the HG film is 36.5°C and the temperature at the upper HG-water interface is 34.4°C (Supplementary Fig.13d). Even though the temperature at the HG-water interface increases (a small amount) during the HG collapse, we also note that the distance between the upper surface of the HG film and the basal plane of the cells increases. These results indicate two favorable characteristics: 1) the temperature inside the HG film is always lower than temperature at the basal plane of the cell (allowing actuation of the microstructures without compromising the physiological conditions) and 2) there is positive feedback in the HG film heating (the temperature inside the HG film rises when it collapses, further promoting the collapse). We also note that in reality the volumetric heat flux Q is not

uniform, but instead has a Gaussian profile due to the laser beam. This results in stronger heating at the mid-plane of the HG film (where the beam is focused), while reducing the temperature at the upper HG interface.

The simulations also yield interesting results on the time-scales of the HG heating and microstructure actuation. Whereas the actuation time scale is on the order of 1 s, the temperature of the HG film reaches close to the steady-state value in time scales that are on the order of milliseconds (Supplementary Fig.13e). This suggests that the actuation time scale is not limited by the heating process, but instead by the dynamics of the HG collapse (reconfiguration of the polymer network and removal of the water), and of the microstructure actuation (Fig.5, Supplementary Fig.9).

Supplementary Note 11. Analysis of cell adhesion to the HAIRS material and the material parameters that determine cell alignment patterns

We plated the D1 ORL UVA cells on the HAIRS-*x*NR samples by a typical cell culture procedure, with the exception that we maintained the temperature of the samples always between room temperature and 31°C. With otherwise typical culture conditions (standard tissue culture dishes), the low temperature growth conditions decrease the cell growth rate (Supplementary Fig.14). Lower than physiological cell growth temperatures have been observed to slow the growth rate for many different cell types, but the cells remain metabolically active, and these conditions are not known to be seriously disruptive to cells in other ways.[7-9]

The cells were incubated on the HAIRS-xNR samples at 31°C, which is just below the LCST of the PNIPAAm, so that the hydrogel remained in its swollen and non-adhesive state during the cell culture period.[10,11] By design, the HAIRS-*x*NR samples therefore presented an attachment surface to the cells with a micron-scale pattern of adhesive and non-adhesive regions, defined by the positions of the microplate tips emerging from the hydrogel layer. Because the cells are only able to attach and form focal adhesion complexes on the microplate tips (Fig.6a, Supplementary Fig.15), which are interspersed in the non-adhesive hydrogel, the spatial arrangement of the microplates therefore provides the opportunity to control the cells' positions on the HAIRS-*x*NR surface, and also their alignment direction if the available adhesive area is specifically confined in one direction (Fig.6, Supplementary Fig.20). By plating the cells on HAIRS-*x*NR samples with columns of microplates that offered between 10–30μm of adhesive area in the lateral direction, and that were spaced apart by at least 10μm, we were able to align the cells along a single axis that coincided with the bending direction of the microplates (Supplementary Fig.16). Importantly, in these growth conditions characterized by a highly patterned surface and a low growth temperature (31°C), the cells were able to divide and migrate over the course of several days until they reached confluency (Supplementary Fig.17). The fact that the PNIPAAm hydrogel was in its swollen and non-adhesive state during these experiments is confirmed by the cross-sectional views of the reconstructed 3D confocal image (Fig.6), and also by the fact that the cells are not seen to attach either to the large stripes of hydrogel between the columns of microplates (Supplementary Fig.16), or to the broad area of hydrogel surrounding the entire microstructure array (Supplementary Fig.17).

The highly reproducible cell alignment patterns on the HAIRS-*x*NR samples with the low temperature culture conditions (Figs.6,7, Supplementary Figs.16,17) is due to the micron-scale adhesive pattern on the surface, defined by the positions of the microplate tips interspersed in the non-adhesive PNIPAAm hydrogel layer. When the cells are grown on the microstructure array substrates without the PNIPAAm hydrogel, the cells align with the microplate long axis unless the flat regions between the columns of microstructures become very wide (Supplementary Fig.18). However, the topography of the microstructure array substrates used for the HAIRS-*x*NR samples without the PNIPAAm hydrogel layer is considerably different than the topography of the HAIRS-*x*NR samples, as the swollen hydrogel fills up the volume from the basal plane to the tips of the microstructures (Fig.6). As a result, the microplates of the same dimensions give a much more significant contact guiding cue in these experimental conditions, and also provide vertical contact planes to the cells. We therefore performed an additional control where we plated the cells on microstructure array substrates with the same lateral dimensions as the HAIRS-*x*NR microstructure arrays, but with 2μm feature heights (Supplementary Fig.19). In these conditions, the cells also align with the long axis of the microplates until the flat regions between the columns of microplates reach a certain width, in which case they tend to grow along the flat regions. These two controls demonstrate that the microstructures and the topography of the HAIRS-*x*NR samples are not responsible for the cell growth and alignment patterns. The PNIPAAm hydrogel is therefore a key component behind the observed behavior.

PNIPAAm hydrogels become adhesive to cells above the LCST at 32°C, however, the phase change also causes a significant decrease in volume.[10-12] In the HAIRS material, this change in volume in turn causes the bending actuation of the microstructures. As a result, the HAIRS-*x*NR samples incubated at 37°C provide a very different type of growth surface to the cells than when they are incubated at 31°C: in addition to the change in adhesiveness of the PNIPAAm hydrogel, there is a significant change in the topography of the surface, with the columns of microstructures forming long ridges. We therefore performed a control to verify the impact on the cells of the pattern in surface adhesiveness on the HAIRS-*x*NR samples by fabricating flat samples with an identical pattern of a non-adhesive PEG functional group (Supplementary Fig.21). Briefly, a thin gold film was deposited on a glass slide, with a patterned photomask masking micro-islands with the same dimensions and positions as the microplates used for the HAIRS-*x*NR samples. The photomask was then removed, and the gold film was functionalized with methoxy-poly(ethylene glycol)-thiol (mPEG-thiol MW ~5000, 5.5mg in 150mL ethanol at ~37°C for 20h) to prevent cell adhesion on the gold film. On these PEG-functionalized samples, the cells attach and align on the surface in the same way as they do on the HAIRS-*x*NR samples incubated below the PNIPAAm LCST, thereby proving that the pattern in surface adhesiveness caused by the presence of the PNIPAAm hydrogel film is responsible for the reproducible cell adhesion and alignment patterns.

Supplementary Note 12. Single cell micro-manipulation with the HAIRS-*x*NR material

In the experiment shown below (Supplementary Fig.22, Movie 11), only a slight stress is applied to the cell at the bottom right of the image. The NIR light was directed to the area on the surface just above the upper edge of the selected cell, and the power was adjusted so that only three rows of microplates supporting the cell were actuated. Under this stimulus, the upper edge of the cell

shifts from the starting position by ~3μm along the surface plane within 4s (Supplementary Fig.22a,c), which is clearly apparent in Movie 7. The maximum local strain is on the order of 17% (Supplementary Fig.22b,d). Since there is no microplate displacement by the fourth row from the edge of the cell, the cell's focal adhesions from that point onward do not experience any applied force. With this set of experimental parameters, the external forces are thus applied only to a small portion of the selected cell. Nonetheless, the cell strain, combined with the slight downward motion of the microplate tips as they bend, still visibly changes the appearance of the cell as the stress propagates inside and causes changes in the overall cell structure and shape. Meanwhile, the neighboring cells are not affected.

From the movies collected during the cell manipulation experiments, images of the cells at specific time points during the experiments may be isolated. Supplementary Figure 23 displays images of several cells taken immediately before and immediately after the start of the experiments (Movies 6-7, 10-11). The images show that the cells resume their initial shape at the end of the experiment. Data from these experiments is also displayed in Figure 7, and Supplementary Figure 22.

We looked for signs of cell mortality after the cell manipulation experiments by returning the cells to full medium in a 31°C incubator (close to initial growth conditions) for 100min, and then staining the samples with Sytox Orange Nucleic Acid Stain according to the manufacturer's protocol. The stain conditions were 2.5μM Sytox Orange in Live Cell Imaging Solution for 10min. The samples were rinsed with Live Cell Imaging Solution, and imaged immediately on a confocal microscope. Figure 7e-h and Supplementary Figure 24 show images of three cells taken from the movies recorded during the mechanical stimulation experiments (Movies 6, 10, 11), and then confocal images of the same cells taken approximately 2h later after the incubation with the Sytox dye. Some of the Sytox Orange dye deposited onto the microstructures, and is useful as it shows their locations. No nuclear staining of any of the manipulated cells with the Sytox Orange was observed. Additionally, one of the cells that were manipulated in the experiment started to migrate over to the neighboring column of microstructures by the time the confocal image was taken (Fig.7g,h). This served as an additional confirmation that the experimental conditions are not cytotoxic in and of themselves.

Supplementary Methods

Supplementary Method 1. Instrumentation

UV-vis measurements of solution samples were performed using a Cary60 spectrometer (Agilent). The absorbance of bulk hydrogel (HG/AuNR) samples was measured on a Benchmark Bio-Rad microplate reader at 350-1000nm range using the Microplate manager 4.0 software. Transmission Electron Microscopy (TEM) images were acquired using a JEOL 2100 microscope (Japan) with an operating voltage of 200kV. The samples for TEM images were made by placing a drop of the AuNRs solution on a TEM carbon-coated grid. High-resolution images were taken using a Gatan Osiris digital camera. Scanning Electron Microscopy (SEM) images were acquired using an FESEM Ultra55 (Zeiss) scanning electron microscope. Gold ion concentrations were determined using an inductively coupled plasma atomic emission spectrometer (ICP-AES) (Massachusetts Materials Research Inc.). The purified AuNR-PEG (0.5mL) were digested by the addition of aqua regia (HNO_3:HCl 1:3, 0.7mL). Gold calibrator solutions were prepared just before analysis by dilution of an aqueous solution of 0.1 M $HAuCl_4$. IR camera SC5600M F/3 (InSb), FLIR Systems ATS (France) has been used for thermal imaging. The photothermal actuation experiments were performed using an NIR laser diode source (Thorlabs, lasing wavelength 808nm) attached to the Olympus BXFM microscope and a Hamamatsu EM-CCD digital camera. The sample O_2 plasma etching step was performed with a Diener Electronic Standard Plasma Systems Femto plasma cleaner. The confocal fluorescence images were taken with a Zeiss LSM 710 confocal microscope.

Supplementary Method 2. Synthesis of PEG-stabilized AuNRs

To obtain 100mL aqueous solution of CTAB capped AuNRs (AuNR-CTAB), seed and growth solutions were prepared as described below.[1] The seed Au nanoparticles aqueous solution stabilized with CTAB has been prepared by mixing of 2.5mL of CTAB solution (0.2M) with 1mL water and 120µL of $HAuCl_4$ solution (15mM). Next, 500µL of freshly prepared, ice-cold $NaBH_4$ aqueous solution (10mM) was added followed by stirring for 2min. The stirring was stopped yielding a brownish yellow dispersion containing ~2nm Au nanoparticles stabilized by CTAB surfactant. To grow AuNR, the growth solution was prepared by addition of 40mL DI water, 5mL of $HAuCl_4$ solution (15mM) and 4mL of $AgNO_3$ (4mM) to 54mL of CTAB solution (0.2mM). Then 1.25mL of ascorbic acid (2mM) was added. The flask was hand-stirred until the mixture became colorless. The obtained growth solution was used right after its preparation. At this point, 1mL of Au nanoparticles seed solution was added to the entire growth solution and the mixture was stirred for 30s. Next, the flask containing the growth solution was placed into a water bath at 27°C for over 18h without any stirring. A reddish-brown color slowly developed within the first 10min indicating the formation of CTAB-stabilized AuNRs to be used in the next step.

PEG-modified AuNR were prepared as follows. A solution of as prepared AuNR-CTAB was centrifuged at 14000rpm for 30min, decanted, and re-suspended in ~20mL of water to remove the excess of CTAB. mPEG-SH (300mg) was dissolved in 3mL water and added to the AuNR-CTAB solution. The mixture was stirred for 24h at room temperature. In the next step, AuNR-

mPEG dispertion was centrifuged at 14000rpm for 30min, decanted, and re-suspended in ~10mL of water to remove the excess of mPEG-SH. This washing procedure was repeated 3x. The last precipitate was redispersed in DMSO, washed additional 3x (14000rpmx30min) and suspended in 7mL of DMSO to give AuNR-mPEG stock solution. The UV-vis absorption spectra of AuNRs exhibit two characteristic surface plasmon bands: longitudinal (~800nm) and transversal (~515nm) (Supplementary Fig.1). The modification of AuNR-CTAB (λ_{max} ~790nm) with PEG-SH resulted in red shift of ~12nm. The transfer of AuNR-PEG from water to DMSO solution resulted in further red shift of ~30nm (AuNR-PEG in DMSO λ_{max} ~840nm). The red shift can be attributed to the change in the dielectric constants of the solvent medium (water ~80 vs. DMSO ~47).

Supplementary Method 3. Fabrication procedure for the microstructure arrays

Silicon masters of the microstructure arrays were fabricated using the Bosch process as described elsewhere.[13,14] The microstructures were rectangular microplates arranged in rectangular arrays (Supplementary Fig.6). In the array, the microstructure dimensions and spacing were varied incrementally in order to test multiple different conditions (36 total). Thus, the microplates were all 12μm-high and 2μm-wide, and the length was varied from 5-30μm with incremental increases of 5μm. The columns of microplates were separated by distances ranging from 5-30μm with incremental increases of 5μm. The rows of microplates were separated by 5μm, which remained constant throughout the entire array. The entire area of this microstructure array covered 0.36cm^2, with each condition remaining constant over 1mm^2. The surfaces of silicon masters were modified by exposing them to (tridecafluoro-1,1,2,2-tetrahydrooctyl)trichlorosilane in a desiccator under vacuum at room temperature for at least 24h to facilitate subsequent demolding steps (of the PDMS). To create negative replicas of the silicon structures, PDMS prepolymer in a 10:1 (wt./wt.) ratio of base resin:hardener was mixed for 3min and degassed under vacuum at room temperature. The PDMS prepolymer mixture was then poured over the silanized silicon master in a petri dish, put under vacuum to remove residual bubbles, and cured for 8h at 70°C. After cooling, the silicon master was demolded by carefully peeling it off the PDMS mold. To make epoxy replicas of the original master, a single droplet (~5μl cm^{-2}) of degassed, room temperature Norland 61 epoxy prepolymer mixture was placed on the 0.36cm^2 patterned area on the PDMS mold. A freshly piranha-treated and oven-dried 1cm^2 glass coverslide piece was put over the prepolymer drop to serve as a flat backing piece in order to ultimately yield flat samples with reproducible areas of 1cm^2. Samples prepared by this method were then cured under a UV lamp (Dymax Light Curing Systems, with a metal screen to reduce the light intensity to ~13mW cm^{-2} at 365nm) for about 45min, during which time the samples and molds were flipped several time to insure even curing of the prepolymer layer and thus minimize the internal stresses in the hardened polymer. The samples were aged at 50°C for 16-18h, allowed to cool to room temperature, and then the samples were carefully removed from the PDMS molds to yield arrays of polymer structures that replicated the original silicon master. Scanning Electron Microscopy (SEM) images were made using FESEM Ultra55 (Zeiss).

Supplementary References:

1 Nikoobakht, B. & El-Sayed, M. A. Preparation and Growth Mechanism of Gold Nanorods (NRs) Using Seed-Mediated Growth Method. *Chem. Mater.* **15**, 1957-1962, (2003).

2 Orendorff, C. J. & Murphy, C. J. Quantitation of metal content in the silver-assisted growth of gold nanorods. *J. Phys. Chem. B* **110**, 3990-3994, (2006).

3 Shiotani, A., Mori, T., Niidome, T., Niidome, Y. & Katayama, Y. Stable incorporation of gold nanorods into N-isopropylacrylamide hydrogels and their rapid shrinkage induced by near-infrared laser irradiation. *Langmuir* **23**, 4012-4018, (2007).

4 Pan, G., Guo, Q., Cao, C., Yang, H. & Li, B. Thermo-responsive molecularly imprinted nanogels for specific recognition and controlled release of proteins. *Soft. Matter.* **9**, 3840, (2013).

5 Kim, P., Zarzar, L. D., He, X., Grinthal, A. & Aizenberg, J. Hydrogel-actuated integrated responsive systems (HAIRS): Moving towards adaptive materials. *Curr. Opin. Solid St. Mat.* **15**, 236-245, (2011).

6 Zarzar, L. D. *et al.* Direct writing and actuation of three-dimensionally patterned hydrogel pads on micropillar supports. *Angew. Chem. Int. Ed.* **50**, 9356-9360, (2011).

7 Jat, P. S. & Sharp, P. A. Cell-Lines Established by a Temperature-Sensitive Simian Virus-40 Large-T-Antigen Gene Are Growth Restricted at the Nonpermissive Temperature. *Mol. Cell. Biol.***9**, 1672-1681 (1989).

8 Watanabe, I. Effects of Temperature on Growth Rate of Cultured Mammalian Cells (L5178y). *J. Cell Biol.* **32**, 309-323, (1967).

9 Hicok, K. C. *et al.* Development and characterization of conditionally immortalized osteoblast precursor cell lines from human bone marrow stroma. *J. Bone Miner. Res.* **13**, 205-217, (1998).

10 Kuroki, H., Tokarev, I. & Minko, S. Responsive Surfaces for Life Science Applications. *Annu. Rev. Mater. Res.***42**, 343-372, (2012).

11 Takezawa, T., Mori, Y. & Yoshizato, K. Cell Culture on a Thermo-Responsive Polymer Surface. *Bio/Technology* **8**, 854-856, (1990).

12 Schmaljohann, D. Thermo- and pH-responsive polymers in drug delivery. *Adv. Drug Deliv. Rev.* **58**, 1655-1670, (2006).

13 Krupenkin, T. N., Taylor, J. A., Schneider, T. M. & Yang, S. From Rolling Ball to Complete Wetting: The Dynamic Tuning of Liquids on Nanostructured Surfaces. *Langmuir* **20**, 3824-3827, (2004).

14 McAuley, S. A. *et al.* Silicon micromachining using a high-density plasma source. *J. of Phys. D-Appl. Phys.* **34**, 2769-2774, (2001).
